# Continuous-surface 3D reconstruction from kilometer-range single-photon LiDAR using score-based priors

**DOI:** 10.1038/s41598-026-57483-5

**Published:** 2026-06-28

**Authors:** Abderrahim Halimi, Jean-Yves Tourneret, Aongus McCarthy, Ewan Wade, Rachael Tobin, Jorge Garcia-Armenta, Phil Soan, Gerald S. Buller

**Affiliations:** 1 School of Engineering and Physical Sciences, Heriot-Watt University, Edinburgh, UK; 2 University of Toulouse, Toulouse, France; 3 Defence Science and Technology Laboratory, Porton Down, Salisbury, SP4 0LQ UK

**Keywords:** Engineering, Mathematics and computing, Optics and photonics

## Abstract

Three-dimensional (3D) imaging underpins applications ranging from autonomous navigation to defense and biomedicine, with single-photon avalanche diode (SPAD) light detection and ranging (LiDAR) enabling fast, long-range, and photon-efficient depth sensing. In practice, reconstruction quality is constrained by limited sensor resolution, particularly in the short- and medium-wave infrared, as well as sensor dark counts, background illumination, atmospheric effects, and motion. We introduce a unified framework for continuous-surface 3D scene representation that integrates multimodal sensing with score-based priors on latent variables. The proposed approach models scenes using a parametric continuous surface, enabling robust rendering at arbitrary spatial resolutions, even in the presence of multiple depth layers allowing imaging through camouflage. We demonstrate capabilities including data compression, targeted high-resolution rendering, and guided super-resolution of dynamic 3D videos. Validated across multiple sensing scenarios using diverse off-the-shelf priors, this framework enables compressed, high-fidelity 3D imaging in real-world environments.

**Supplementary Information:**

The online version contains supplementary material available at https://doi.org/10.1038/s41598-026-57483-5.

## Introduction

Three-dimensional (3D) imaging plays a critical role in a wide range of applications, including autonomous navigation, environmental monitoring, robotics, and augmented reality. Among the various 3D sensing technologies, single-photon LiDAR (SPL) has emerged as a powerful tool due to its unique ability to perform depth imaging with exceptional precision and surface-to-surface resolution^1–3^. SPL systems are based on time-correlated single-photon counting (TCSPC), where laser pulses illuminate the scene and single-photon detectors, such as SPAD arrays, measure the time-of-flight (ToF) of the reflected photons with picosecond resolution. This allows for precise reconstruction of both static and dynamic scenes, even in photon-starved conditions. Notably, SPL has demonstrated robust performance in long-range imaging (up to several kilometers)^4–6^, imaging through camouflage netting or highly scattering environments (such as fog or underwater)^7–9^, determining arboreal parameters^10^, and targets in cluttered scenes such as foliage^11^. The combination of low-light sensitivity, high temporal resolution, and recent advances in SPAD array technology^12^ makes SPL a leading candidate for full-field 3D imaging across diverse and challenging scenarios.

Despite its advantages, SPL faces several challenges in real-world applications. The detection of photon-level events across multiple dimensions (spatial, temporal, and spectral) results in large data volumes that are difficult to store, transmit, and process efficiently. These challenges are compounded when scenes are observed using multiple complementary sensors, requiring effective multimodal data fusion. Additionally, SPL array data is often low-resolution in the XY plane due to sensor limitations, and can be blurred by pixel cross-talk or scene dynamics, leading to the presence of multiple surfaces within a single pixel footprint. Conventional voxel-based or event-based representations^13,14^ are memory-intensive, while image-based reconstructions that assume a single surface per pixel^15,16^ are insufficient for complex or cluttered environments. Therefore, there is a critical need for compressed data representations that preserve scene structure and support flexible rendering. Moreover, guided super-resolution algorithms are required to enhance resolution, remain robust to noise, and leverage multimodal cues, enabling accurate 3D reconstruction in challenging conditions.

Computational reconstruction and super-resolution methods can be broadly categorized by their underlying data representation. Early methods typically operate on image-based representations that assume a single surface per pixel. These include model-based approaches relying on statistical inference or optimization^15,16^, as well as learning-based methods trained on paired low- and high-resolution images^17^. While these approaches are computationally efficient, they often fail in complex scenes with multireflection per pixel. To address such limitations, a second family of methods processes raw SPL data, such as histograms of counts or event detections, preserving the full spatio-temporal information. Statistical approaches such as MANIPOP^18^ and M2R3D^19^ model the 3D photon distributions to recover multiple surfaces per pixel. Deep learning techniques have also been developed for histogram-based reconstruction^20^, but often require extensive labeled data and are sensitive to training conditions. A third class of approaches focuses on compressed representations to overcome the growing size of high-dimensional SPL data. These include point cloud representation^21^, sketched representations^22^, multiscale models^8,23^, and compressive networks^24,25^. Adopting such representations within hybrid approaches, at the interface of optimization and deep-learning, have demonstrated efficiency. Examples include the point-cloud-based plug-and-play algorithm^21^, and the multiscale unrolled networks^26–28^. More recently, continuous scene representations with neural fields have gained attraction, as they learn implicit functions to represent 3D scenes at arbitrary resolutions, rather than as discrete sets of data points^29^. Among them, Gaussian splatting^30^ has shown impressive rendering results in addition to enhanced interpretability. However, these methods remain scene-dependent, and computationally demanding as they often rely on slow pixel-wise rendering, which can be a bottleneck in time-sensitive applications. This landscape of methods highlights the trade-offs between fidelity, interpretability, and scalability that continue to shape 3D imaging and SPL research.

This paper presents a unified framework for compressive and continuous 3D scene representation, enabling flexible rendering with any upsampling factors while leveraging multiple sensing modalities and enhancing robustness to noise through multiscale modeling. The approach adopts a Bayesian formulation, where a Gaussian mixture model (GMM) is used as the likelihood to capture the 3D scene density, and state-of-the-art algorithms are incorporated as priors via the score function. The proposed method is validated on a variety of real-world scenarios, demonstrating improved resolution and surface reconstruction in cluttered environments and during long-range imaging of both static and dynamic scenes. Its performance is further assessed on simulated data for key tasks such as 3D reconstruction, denoising, guided upsampling, and data compression. Lastly, we highlight the framework’s versatility through its successful application to downstream computer vision tasks, with saliency detection as an example.

**Fig. 1:**
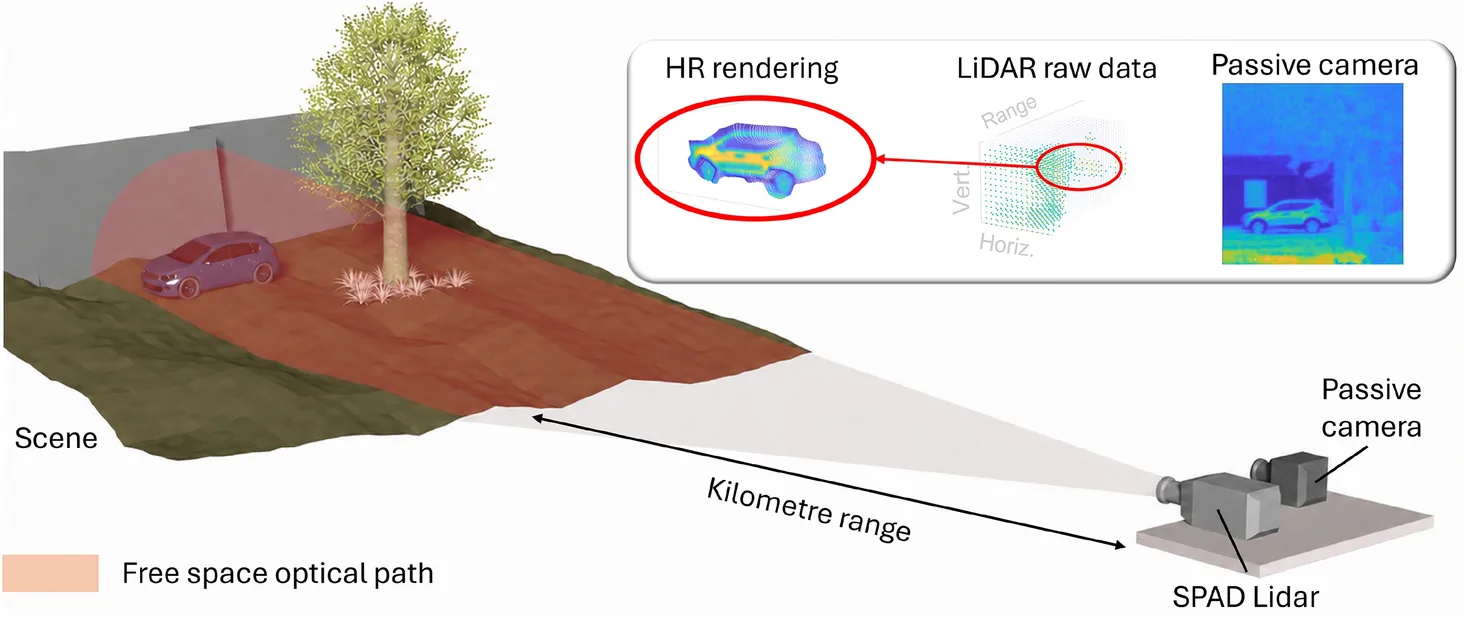
Multi-sensor measurement set-up: the setup contains a bistatic time-of-flight depth imaging system composed of a 32 *×* 32 format InGaAs/InP SPAD detector array, a pulsed fiber laser source, and a passive high-resolution (HR) camera. In the results shown, this single-photon time-of-flight approach was used to obtain depth information of targets at ranges of 1.4 km from the transceiver location. The LiDAR ToF data and HR intensity image are jointly processed by the proposed algorithm to deliver HR 3D reconstructions. The schematic was created by the authors, with AI-assisted refinement of the illustrative car and tree shown on the left-hand side, these two elements do not represent experimental data or results.

## Results

We first evaluate the proposed approach on a series of real-world long-range experiments acquired at a standoff distance of 1.4 km. We begin with the reconstruction of static targets at long distances, including cases with an intensity guide or imaging through camouflage netting. These experiments assess the robustness of the method under severe attenuation and structured occlusions. We then demonstrate the extension of the framework to dynamic settings, where the framework is used to generate temporally consistent 3D video reconstructions. Next, we briefly illustrate how the proposed representation can be leveraged as a compact and informative intermediate representation for downstream vision tasks, highlighting its broader applicability beyond reconstruction. Finally, we provide a controlled analysis on simulated datasets with clean high-resolution ground truth, enabling a systematic evaluation of reconstruction accuracy under well-defined noise levels and photon sparsity conditions. **Computing platform:** All simulations were performed in MATLAB R2024a on a workstation equipped with an Intel(R) Xeon(R) Gold 6230 CPU @ 2.10 GHz and 192 GB RAM, using CPU implementation only. DVSR^31^ was implemented in Python and executed on an NVIDIA GeForce RTX 3090 GPU with 24 GB memory.

### Reconstruction of static scenes at kilometer-range and through camouflage netting

**Fig. 2:**
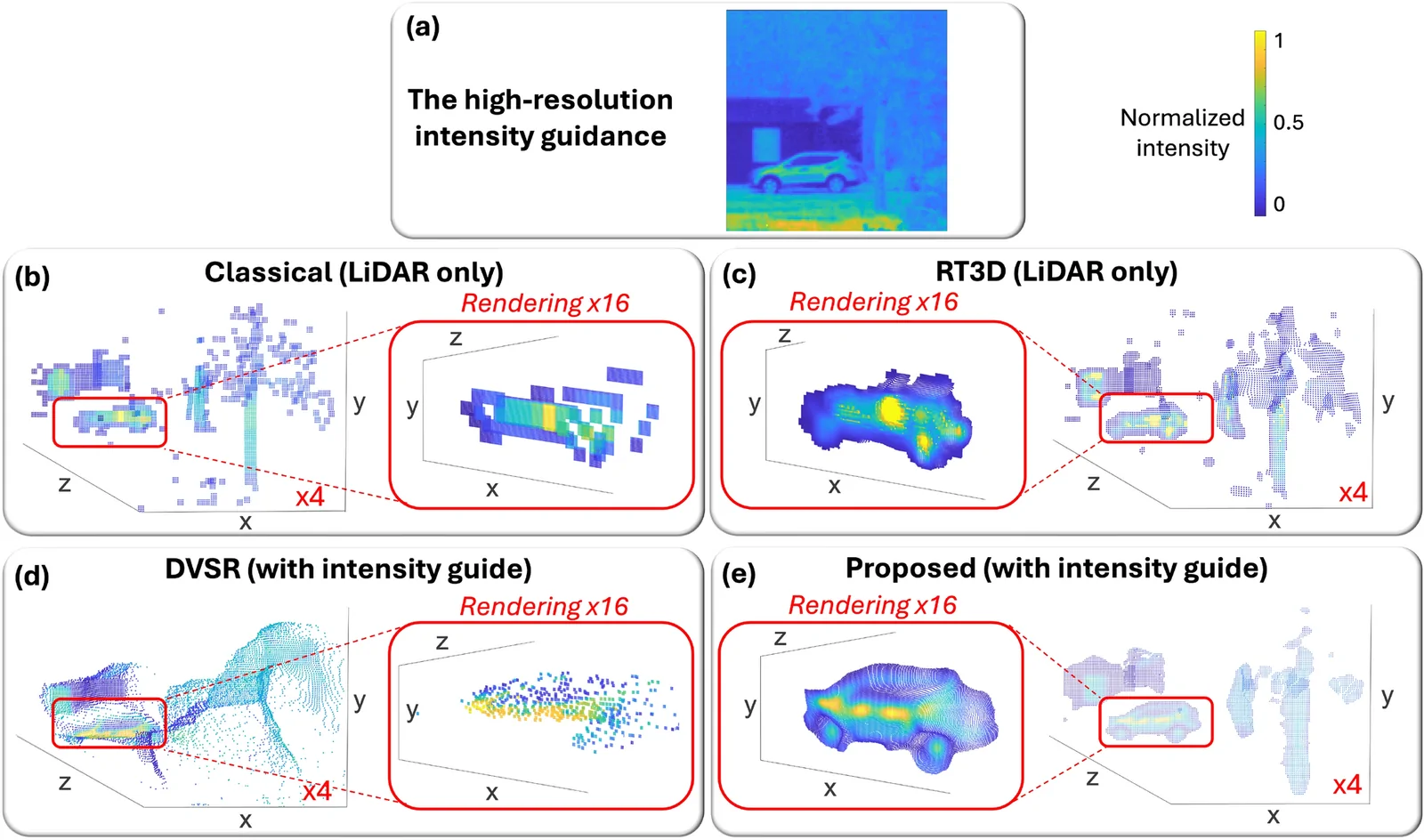
3D upsampling and rendering results for a car scene acquired at 1.4 km stand-off distance using a SWIR camera and a LiDAR system. (**a**) Intensity image of the SWIR camera with *128 × 128* pixels. 3D upsampling results (**b**) using classical MLE nearest interpolation algorithm, (**c**) RT3D algorithm, (**d**) DVSR with intensity guidance and (**e**) proposed GMM algorithm with a DVSR-prior. The proposed algorithm uses the LiDAR data with the upsampling factor $u_f=4$ and intensity image (**a**) to estimate the GMM, that is then used to render different resolutions. We show an upsampled result by *× 4* to obtain an *128 × 128* image, and a *× 16* rendering. The colors encode the normalized intensity estimated by classical, RT3D and proposed, and the reference intensity for DVSR. Images with depth encoded colors are available in the supplementary.

**Fig. 3:**
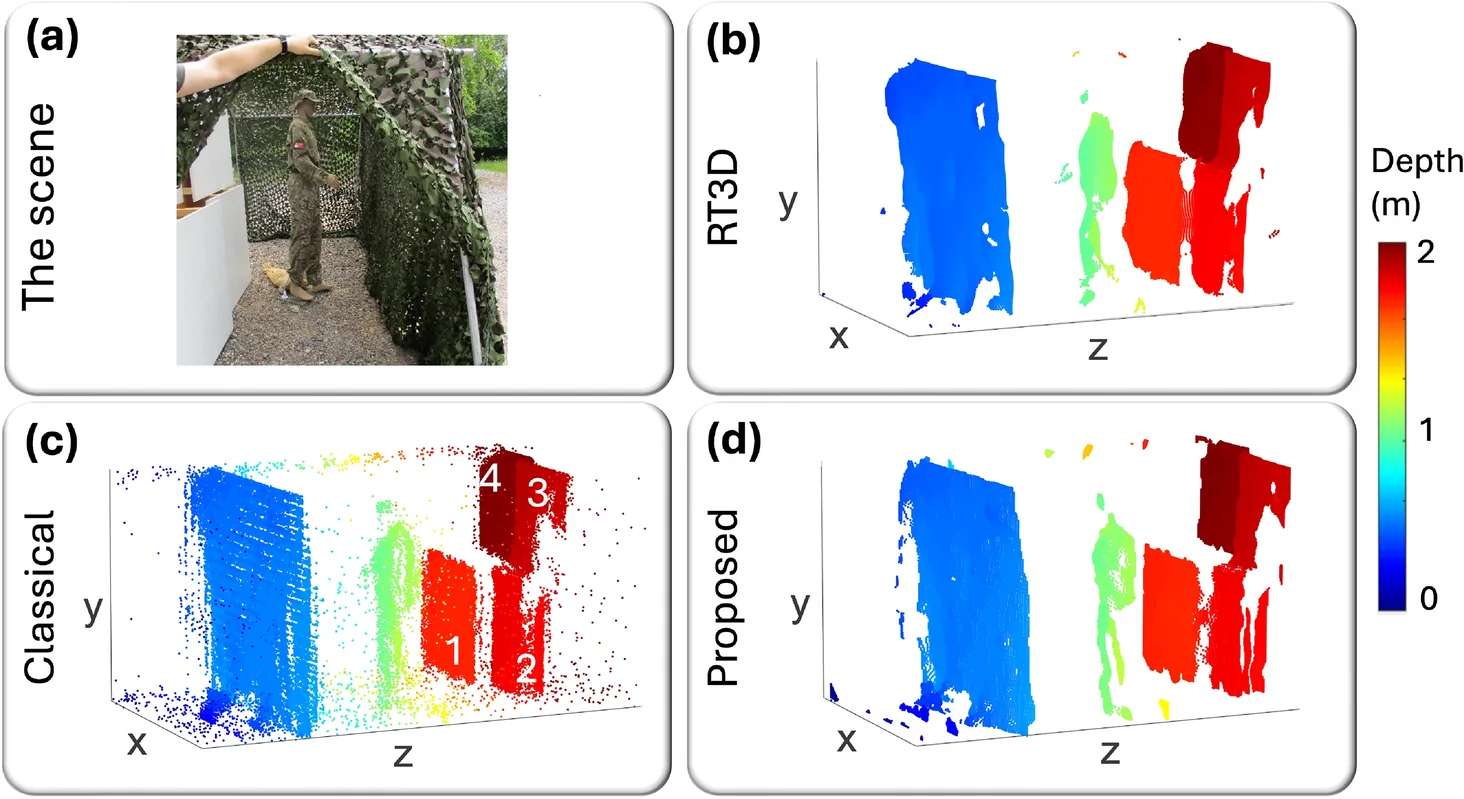
Illustration of the denoising and reconstruction performance of the proposed algorithm in presence of multiple peaks per pixel. The scene is composed of a mannequin and four planar boards behind camouflage netting (**a**) acquired at 1.4 km stand-off distance from the LiDAR system operating with an average laser power of 1.75 mW. Reconstruction results are shown for the RT3D (**b**), classical (**c**), and proposed GMM (**d**) algorithms. The colors encode depth.

**Fig. 4:**
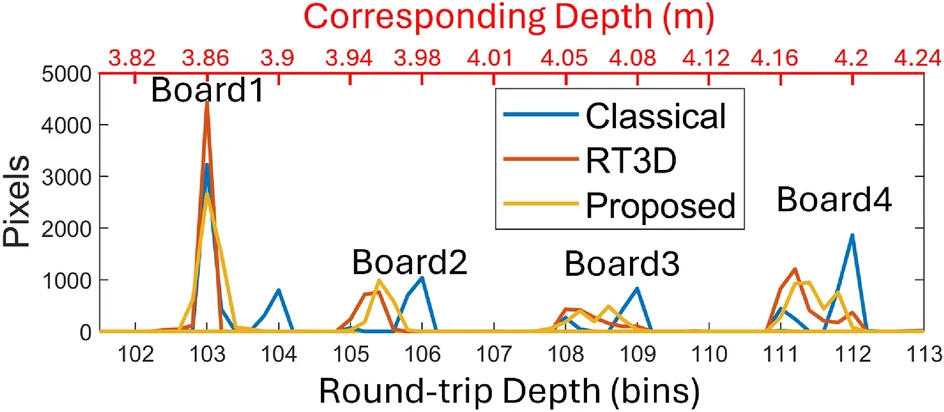
Depth histograms for pixels on the four planar boards obtained using the classical, RT3D, and proposed algorithms. Classical exhibits multiple peaks due to limited range resolution, whereas RT3D and proposed produce more concentrated distributions around the planar surfaces with smaller standard deviations. The x-axis shows the round-trip distance between the surfaces in bins. The histogramming bin width is 250 ps (equivalent to a time-of-flight of 75 mm).

The first scene we consider features a car and a tree in front of a building, acquired at a distance of 1.4 km using a single-photon LiDAR sensor and a short-wave infrared (SWIR) passive sensor as illustrated in Fig. 1. The LiDAR data has 793 temporal bins each representing 250 ps (i.e., a time-of-flight distance of 7.5 cm which is equivalent to the round trip distance for a depth of 3.75 cm). It operates with an average optical power of 220 mW which yielded estimated noise levels of average photons-per-pixel PPP*=36610* counts and signal-to-background ratio SBR*=0.01*. The passive camera had *128 × 128* pixels covering the field of view (i.e. *× 4* spatial resolution compared to LiDAR) and is used as guidance in the proposed reconstruction algorithm. Fig. 2 compares the Classical maximum likelihood estimation (MLE) (b), the plug-and-play-based (RT3D)^21^ (c), the depth video super-resolution (DVSR)^31^ (d), and proposed (e) algorithms when upsampled by factor *× 4* and rendered at *× 16*. In what follows, we use the term *upsampling* when increasing the resolution by a factor $u_f$ to match the size of the intensity guidance, and the term *rendering* when considering larger factors. The proposed algorithm used a number of Gaussian components *M=1280* Gaussians, and a point cloud denoiser to approximate the prior’s score when estimating the GMM parameters. This estimation required approximately 52 seconds using the MATLAB CPU implementation described in the Discussion Section. This representation enables rendering at any resolutions (hence *× 16* rendering) and within any specified region of interest, in contrast, both RT3D and DVSR require a new run and modification of the input data to generate the desired upsampled results. Results with *× 4* are blocky for the classical, oversmoothed with DVSR, and contain outliers for RT3D, with the best performance obtained with the proposed strategy. This is highlighted when considering the *× 16* rendering results where classical and DVSR produce fragmented and noisy surface reconstruction. Note that RT3D offers better performance but could not retrieve small features as it is not using an intensity guide. In contrast, the proposed approach yields a high-quality, outlier-free point cloud, accurately capturing the shape of the vehicle in the scene. This demonstrates the advantages of the proposed model-based framework for multiscale continuous scene representation. The second scene contains a human mannequin and four planar boards (spaced at depth intervals of 100 mm) placed behind camouflage netting, captured from a distance of 1.4 km using the *256 × 256* pixels microscanning LiDAR system described in the Experimental setup section  (microscanning with a *32 × 32* detector array). The resulting histogram has 192 temporal bins each corresponding to 250 ps. The 1550 nm wavelength system operates at eye-safe average optical powers of 105 mW, 14 mW, and 1.75 mW, where lower powers result in decreased signal-to-noise if acquisition time is the same. We consider the 1.75 mW leading to an estimated noise levels PPP *≈ 2000* counts and SBR *≈ 0.023*. Fig. 3 shows a picture of the scene (a), and the reconstruction results of the classical MLE assuming up to two peaks per pixel (c), the RT3D (b) and proposed algorithm for *M= 1280* Gaussians and using a point cloud denoiser to approximate the prior’s score (d). The reconstruction time of the proposed algorithm was approximately 103 seconds using the MATLAB CPU implementation described in the Discussion Section. In contrast to the classical algorithm showing noisy results, both RT3D and proposed algorithms reconstruct a cleaner and more continuous representation of the scene indicating their ability to reconstruct multi-layer scenes. This observation is supported by an analysis of the depth distributions for pixels corresponding to the four planar boards. Fig. 4 presents the depth histograms obtained using the classical, RT3D, and proposed algorithms. Since each board is a continuous planar surface, an accurate reconstruction should produce a single peak, which is narrow when the board is approximately perpendicular to the system line of sight, and wider when the board is slanted. The classical method shows a discontinuous planar surface resulting in multiple peaks, reflecting its limited range resolution even when using the center-of-mass method. In contrast, RT3D and the proposed method exploit spatial correlations between pixels to estimate more consistent planar surfaces, leading to a single connected peak as expected for a continuous planar surface. Table 1 further quantifies this observation by reporting the standard deviation (STD) of the residuals after fitting a planar surface to each reconstructed board. Lower values indicate that the estimated point cloud is closer to a planar surface. The lower STDs obtained by RT3D and the proposed method therefore confirm their improved depth consistency compared to the classical method. We finally note the absence of a high-resolution (HR) intensity guidance due to imaging through camouflage netting, however, the next section illustrates the performance when imaging a moving person with intensity guidance.

**Table 1:** Depth STDs (in bins) on the four planar boards computed for each algorithm after fitting a planar surface to the corresponding estimated point cloud.

No.	Boards			
	*#1*	*#2*	*#3*	*#4*
Pixels	4600	1740	1472	3186
Classical	0.84	0.19	0.27	0.33
RT3D	0.06	0.07	0.12	0.13
Proposed	0.07	0.11	0.10	0.07

### Guided super-resolution of 3D videos

**Fig. 5:**
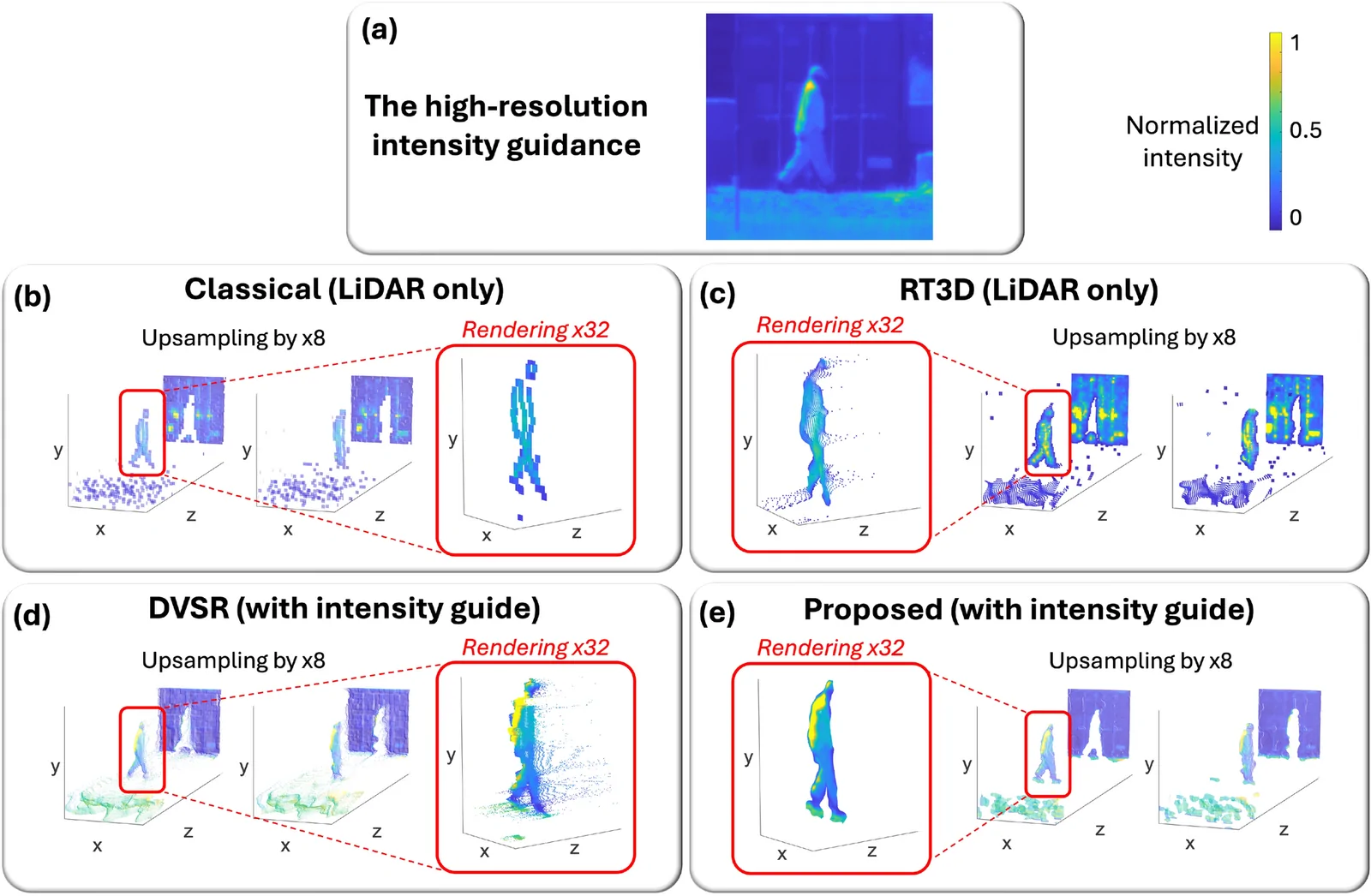
Illustration of the reconstruction and rendering performance of the proposed algorithm when processing a video. The scene is composed of a walking man acquired at 1.4 km stand-off distance using a SWIR camera and a LiDAR system. (**a**) Intensity image of the SWIR camera with *256 × 256* pixels. 3D upsampling results (**b**) using classical nearest interpolation algorithm, (**c**) RT3D algorithm, (**d**) DVSR with intensity guidance and (**e**) proposed GMM algorithm with a DVSR-prior. The proposed algorithm uses the LiDAR data with the upsampling factor $u_f=8$ and intensity image (**a**) to estimate the GMM, that is then used to render different resolutions. We show an upsampled result by *× 8* to obtain a *256 × 256* image, and a *× 32* rendering. The colors encode the estimated normalized intensity for classical, RT3D and proposed, and the reference normalized intensity for DVSR. A video representation of this scenario is provided in Movie S1.

We consider a scene featuring a person moving in front of a shipping container, acquired at a standoff distance of 1.4 km using two sensors operating at frame rates exceeding 20 fps. The 1550 nm wavelength LiDAR system, which recorded photon arrivals in 326 temporal bins of 250 ps each, operated at an eye-safe average optical power of 38 mW, yielding estimated noise levels of PPP *≈ 1400* counts and SBR *≈ 0.06*. The passive camera had *256 × 256* pixels covering the field of view (i.e. *× 8* higher resolution compared to LiDAR). Thanks to the high sensitivity of the SPAD array and the robustness of the depth extraction algorithm, multiple depth layers can be clearly resolved, even in high-background lighting conditions exceeding 100 klux (i.e. full sunlight). A sequence of six video frames was processed, requiring 2118 seconds for the proposed algorithm. Two *× 8* upsampled representative outputs (frame 1 and 6) are shown in Fig. 5 when using the studied algorithms. This figure also shows a *× 32* rendering of the person (rendered using the estimated GMM of the proposed algorithm, whereas other methods require re-running with updated input data). The classical algorithm in Fig. 5 (b) exhibits blocky reconstructions of the human subject, with disconnected depth layers and noisy surface geometry. The RT3D (c) and DVSR (d) show better results although they over-smooth the scene leading to a bleeding effect joining disconnected surfaces. In contrast, the proposed method, combined with the DVSR algorithm to approximate the prior-score function, produces a continuous scene representation that enables rendering at any resolutions and within any chosen region of interest. Fig. 5 (e) presents the corresponding depth and reflectivity estimates showing a clean, outlier-free point cloud that faithfully captures the human shape and ensures temporal consistency across frames, due to the spatio-temporal priors enforced by the DVSR model. These results highlight the significant advantages of the proposed framework over conventional frame-wise processing.

### Other applications: compressed representation for vision tasks

**Fig. 6:**
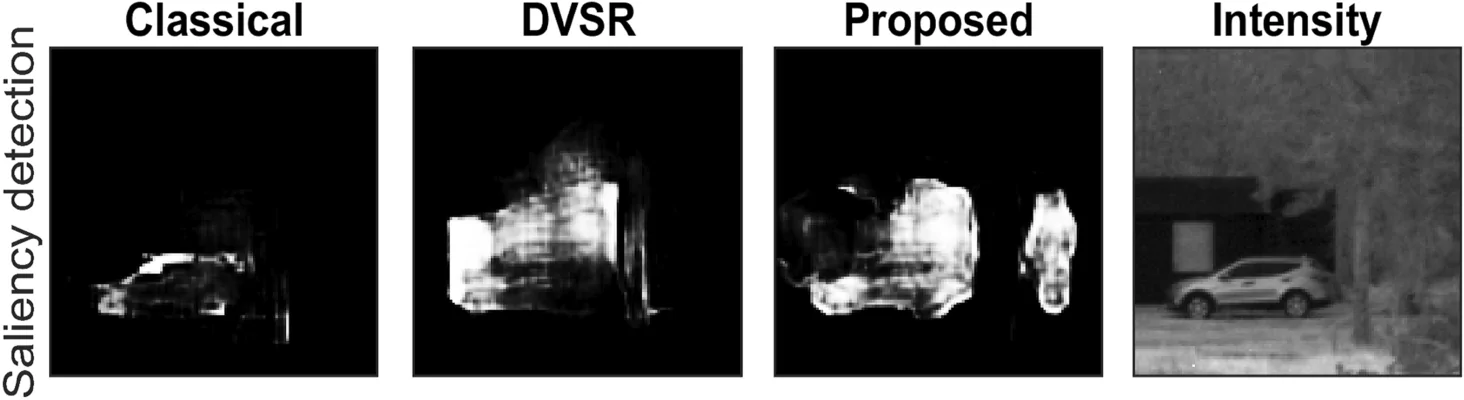
Use of the proposed representation for downstream vision tasks: examples of binary saliency detection maps using UCNet^32^ when considering the reference intensity map and depth maps estimated by the classical MLE, DVSR and proposed algorithms.

The proposed approach provides a compressed data representation that enables high-level 3D vision tasks by leveraging 2D priors, such as 3D pose estimation, 3D scene segmentation, or 3D object tracking. As an example, we use the reconstructed high-resolution depth and the reference intensity image (for a fair comparison with DVSR that does not estimate intensity) to perform saliency detection with UCNet^32^, enabling the identification of regions of interest useful for targeted rendering. Fig. 6 shows the saliency maps for the car scene obtained using the classical MLE, DVSR, and the proposed method. The proposed approach provides clearer detection of the car and tree regions, benefiting from its more accurate depth estimation. Other potential applications are beyond the scope of this paper and are left for future work.

### Analysis on simulated dataset

This section evaluates the performance of the proposed algorithm on three tasks: 3D reconstruction under varying noise levels, super-resolution with multiple upsampling factors, and data compression using different numbers of Gaussians. Simulations are based on the Art scene from the Middlebury dataset (Available at: http://vision.middlebury.edu/stereo/data/)^33^, since it features complex structure and is commonly used to evaluate algorithms^16,20,25,26,34^. The luminance of the RGB image is used to generate a *256 × 256* intensity map ${\boldsymbol{r}}=\{r_{x,y}\}$, which is combined with the depth map ${\boldsymbol{d}}=\{d_{x,y}\}$ to simulate photon events. The photon count $c({\boldsymbol{p}})$ at voxel ${\boldsymbol{p}}= [x,y,z]$ is simulated according to a Poisson distribution^8,16^

$$\begin{aligned} c({\boldsymbol{p}}) \sim \mathscr {P} \left[ r_{x,y} f_{\textrm{irf}}(z - d_{x,y}) + b_{{\boldsymbol{p}}} \right] , \end{aligned}$$

where $f_{\textrm{irf}}$ is a Gaussian-shaped impulse response, $b_{{\boldsymbol{p}}}$ is the background level, and *z ∈ [0, Z]* with *Z = 300* bins. The resulting 3D histogram of counts has dimensions $256 \times 256 \times 300$. Noise conditions are quantified by the average photons-per-pixel $\textrm{PPP} = \frac{1}{XY} \sum _{x,y} (r_{x,y} + \sum _z b_{{\boldsymbol{p}}})$ and signal-to-background ratio $\textrm{SBR} = \sum _{x,y} r_{x,y} / \sum _{x,y} b_{{\boldsymbol{p}}}$. Histogram downsampling by a factor $u_f$ is performed by filtering histogram cubes using a $u_f \times u_f$ uniform filter followed by subsampling with a factor $u_f$ in the X and Y directions. To define the score prior, the proposed algorithm employs a simple model-based point cloud denoiser, applying outlier rejection (e.g., pcdenoise in MATLAB) and then Gaussian smoothing.

**Fig. 7:**
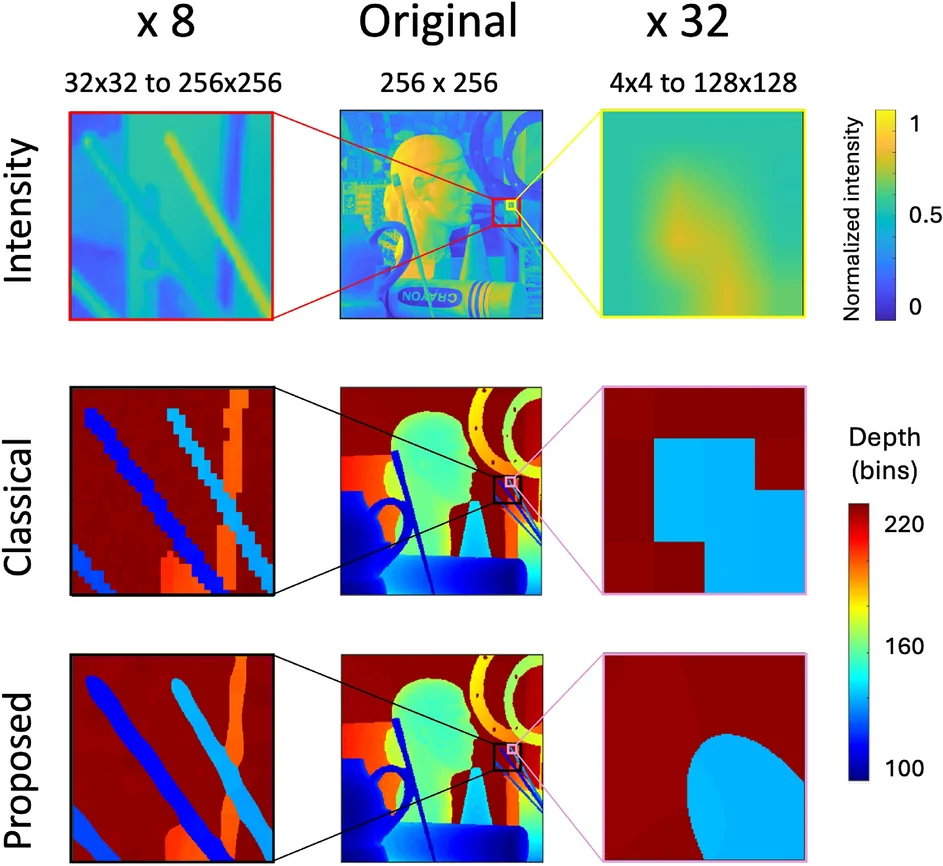
Illustration of the reconstruction and rendering performance of the proposed algorithm on the Middlebury Art scene. (Top row) Intensity image, (Middle row) depth reconstructed using the classical algorithm, (bottom row) depth reconstructed using the proposed algorithm. (Middle column) native *256 × 256* pixels, (left column) rendering by *× 8* of a paint brush area of size *32× 32* pixels in the native resolution, (right column) rendering by *× 32* of a tip of a paint brush of size *4× 4* pixels in the native resolution. The proposed algorithm uses the LiDAR data with the upsampling factor $u_f=1$ and intensity image (top-middle) to estimate the GMM, that is then used to render different resolutions.

**Fig. 8:**
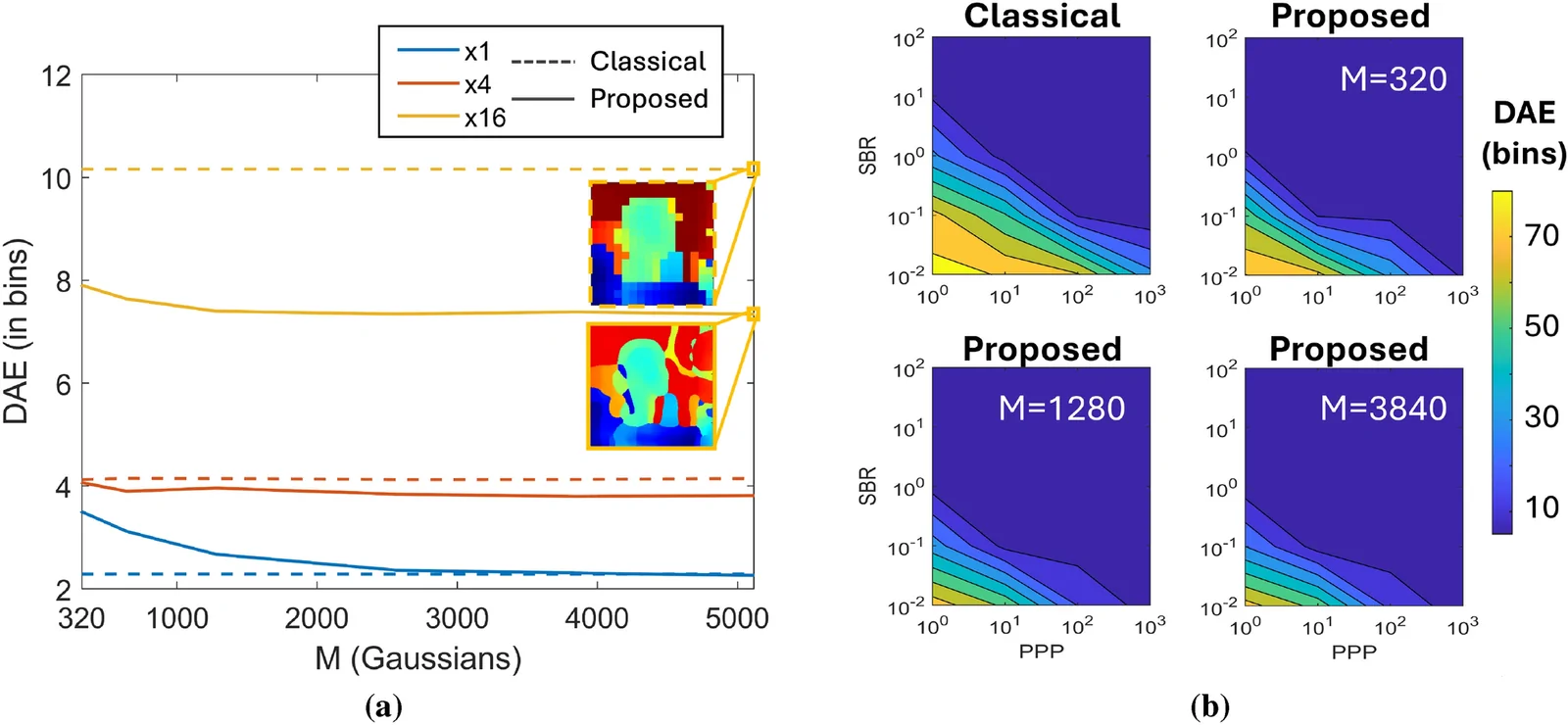
Depth reconstruction accuracy under varying resolution, sparsity, and noise conditions. **(a)** Depth absolute error (DAE) results for the classical and proposed algorithms under (PPP = 1000, SBR = 100), showing effects of varying upsampling factor $u_f$ and number of Gaussians *M*. Depth examples shown for $u_f=16$ (upsampling a *16 × 16* depth image to *256 × 256* pixels). **(b)** DAE results (in bins) comparing the classical and proposed algorithms evaluated over multiple photon sparsity settings (photon-per-pixel, PPP), and noise levels (signal-to-background ratio, SBR). Results obtained for $u_f=1$ and *M=320, 1280* and 3840 Gaussians.

The first experiment illustrates the continuous representation of the scene using simulated data, where the proposed algorithm is used to fit the 3D scene (with *M=5120* Gaussians) and then renders different resolutions. This is achieved by using a high-resolution *256 × 256* intensity guidance (Fig. 7 (top-middle)), and histograms with SBR *= 10*, PPP *= 100*, and $u_f=1$ (i.e. LiDAR has *256 × 256* pixels). Fig. 7 (middle column) shows similar maps for the proposed and classical algorithms at native resolution (*256 × 256* pixels). However, the proposed method enables rendering at any resolutions, as shown in the *× 8* and *× 32* rendered results (left and right columns), which demonstrate improved spatial coherence compared to the classical blocky output. The second experiment evaluates compression by varying the number of Gaussians *M* and upsampling factor $u_f$ under clean histogram conditions (PPP *= 1000*, SBR *= 100*), and *256 × 256* intensity guidance. Fig. 8 (a) shows that the depth absolute error (DAE) increases with lower *M* or higher $u_f$, as expected. Nonetheless, the full $256 \times 256 \times 300$ cube is effectively approximated with just *M = 1280* Gaussians (compressing the $19 \times 10^6$ histogram coefficients to 11, 520 parameters, since 9 parameters are required per Gaussian due to 3 coordinates and a symmetrical covariance matrix). Focusing on depth maps, this yields a compression factor of approximately 5 over storing a depth map of $256^2$ pixels. This factor increases significantly when rendering larger images, reaching 20 when rendering an image of $512^2$ pixels, and 95 when rendering an image of $1024^2$ pixels. An example is provided for $u_f=16$, corresponding to the upsampling of a *16 × 16* depth map to *256 × 256*. In this case, the classical algorithm fails to preserve certain small objects, whereas the proposed method successfully recovers them (see reconstructed cone and rings for example). The third experiment analyzes robustness to noise by varying PPP *∈ [1, 1000]* and SBR *∈ [0.01, 10]*, using *M = 1280* Gaussians and $u_f=1$. Fig. 8 (b) presents DAE for both classical and proposed methods. Although both perform well at high PPP and SBR, the proposed approach outperforms in sparse and noisy conditions, due to its multiscale design (e.g., using a *5 × 5* filtered scale). The figure shows that using a small number of Gaussians, *M=320*, already slightly improves over the classical algorithm, while performance stabilizes for *M ≥ 1280*. This supports the use of *M=1280* as a practical trade-off between reconstruction accuracy and computational cost in the real-data experiments. These experiments quantitatively demonstrate the advantages of the proposed algorithm across various scenarios.

## Discussion

The proposed method delivers a powerful and versatile solution for 3D single-photon reconstruction, enabling user-defined spatial resolutions and targeted rendering of regions of interest. The framework achieves substantial resolution enhancement beyond the native LiDAR sensor capabilities by fusing complementary high-resolution modalities and incorporating prior knowledge through the score function. Although the continuous representation can in principle be rendered at even higher upsampling factors, the physically meaningful details remain limited by the information available in the LiDAR measurements, the resolution of the reflectivity guidance, the number of Gaussian components, and the reconstruction prior. For example, rendering at *× 64* in Fig. 5 would produce denser surfaces but would not recover fine details absent from the measurements or guidance image. Future work will investigate generative priors to synthesize such details while remaining consistent with the measurements. Its multiscale modeling ensures robustness to extreme noise and photon sparsity, making it well-suited for challenging conditions such as kilometer-range imaging, high-speed acquisitions, or imaging through turbid media like fog, water, or camouflage netting. Although the real experiments were conducted at 1.4 km, this distance was chosen as a challenging low-SNR scenario rather than a limitation of the framework. Changes in acquisition range, illumination, and obscurant conditions are captured through the PPP and SBR levels, whose impact on reconstruction accuracy is quantified in Fig. 8. Scattering-induced non-uniform backgrounds are also handled through the background estimation procedure described in the Supplementary Information (Figs. S1–S2). In simulated experiments, the method achieved compression factors exceeding 1000 for high-resolution LiDAR datasets, while in practical scenarios it consistently improved the spatial resolution of low-resolution data when guided by an intensity map. Beyond static reconstructions, the approach has been demonstrated on complex scenes containing multiple signal peaks per pixel, such as camouflage netting, and applied to video sequences, where temporal consistency was enforced through the prior term, enabling high-quality upsampling without introducing motion artifacts. These capabilities open the door to applications ranging from large-scale environmental mapping and long-range surveillance to autonomous navigation and augmented reality.

The current MATLAB implementation is computationally limited, as it does not exploit the inherent parallelism of the algorithm. The computational cost is analyzed in the Supplementary Material by varying the upsampling factor $u_f$, the number of Gaussians *M*, and the photon sparsity and noise levels through PPP and SBR, respectively. A GPU-based parallel implementation, as shown in^21,35^, would enable real-time processing. In addition, a fully adaptive selection of *M* according to scene complexity, photon sparsity, and noise level remains an interesting direction for future work. Additionally, the current framework is restricted to a single wavelength; future work will extend it to handle multispectral LiDAR^8^ and higher-dimensional data such as polarization^36,37^. Beyond LiDAR, this framework has the potential to generalize to other event-based modalities, including fluorescence lifetime imaging and electron spectroscopy.

## Methods

### Experimental setup

The experimental setup, illustrated in Fig. 1, uses multiple sensors to image static and dynamic scenes from kilometer-range stand-off distances. A *32 × 32* InGaAs/InP SPAD detector array (Princeton Lightwave) was used as part of a bistatic direct time-of-flight (dToF) LiDAR system. The system operates at a wavelength of 1550 nm, which offers higher laser eye safety thresholds and reduced background noise compared with LiDAR systems operating in the near-infrared^38^. The SPAD array acquired binary frames at approximately 150k frames per second, which were accumulated into photon-count histograms over time windows matched to the passive camera frames. An eye-safe average optical power of *≤ 220* mW was used to flood illuminate the scene in all the experiments. Simultaneously, passive reference images were captured at 20 frames per second using a *640 × 512* pixel Raptor Ninox 640 SWIR camera, providing intermediate transverse resolution for image guidance and fusion. To increase spatial resolution in some experiments, the native *32 × 32* LiDAR sensor was mounted on Newport X–Y translation stages (UTS50CC) that executed an 8-step microscanning pattern in both the *x* and *y* directions, producing a *256 × 256* composite scan.

### Observation model

A single-photon LiDAR system illuminates a scene with a laser and records the time intervals between laser emission and the detection events of individual target return photons as picosecond resolution time-tags. At each pixel, the TCSPC hardware is used to measure the time-of-flight of each collected photon, allowing it to determine a corresponding 3D location ${\boldsymbol{p}}_i = [x_i, y_i, z_i]$, representing the *i*-th voxel in the scene. This voxel may receive multiple photon counts, which are denoted as $c_i$. The observed photons are modeled as a linear combination of signal photons (those reflected from the target) and background photons (such as ambient light or the detector’s dark counts). In contrast to classical works that model each pixel separately, in this work, we assume that the detected photons are sampled from the following 3D mixture of distributions

1 $$\begin{aligned} {\boldsymbol{p}}_i(c_i) | {\boldsymbol{\Theta }}\sim \sum _{m=1}^{M} \alpha _m \mathscr {N}({\boldsymbol{\mu }}_m, \bar{{\boldsymbol{\Sigma }}}_m ) + \alpha _{M+1} \mathscr {B}({\boldsymbol{p}}_i), \text { subject to } \sum _{m=1}^{M+1} \alpha _m = 1, \end{aligned}$$

which indicates that $c_i$ photons have been detected at the 3D location  ${\boldsymbol{p}}_i$, $\mathscr {N}$ denotes the Gaussian distribution, and $\mathscr {B}(.)$ the background distribution that could be non-uniform in range as observed when imaging in scattering environments such as through fog, or underwater (the background shape is assumed known, as described in the Supplementary). The mixture of Gaussians is defined by the parameters ${\boldsymbol{\Theta }}= \{ {\boldsymbol{\mu }}_m, \bar{{\boldsymbol{\Sigma }}}_m, \alpha _m \}, \quad \forall m = 1, \cdots , M$ including the weights $\alpha _m$, the mean vectors ${\boldsymbol{\mu }}_m$, and the covariance matrices $\bar{{\boldsymbol{\Sigma }}}_m$, which capture the amplitude, position, and orientation of each 3D Gaussian component. Given these parameters, one can represent the continuous volume density of the scene and, consequently, estimate the scene’s point cloud by sampling points from regions of high density. Alternatively, depth maps can be estimated by identifying the range (depth) corresponding to the maximum density at each pixel location, defined by real-valued (*x*, *y*) coordinates.

In this paper, we investigate multimodal imaging, where a scene is captured using a high-resolution passive camera and a low-resolution LiDAR sensor (see Fig. 1). The low spatial resolution of LiDAR results in large pixels with wide footprints, causing each pixel to potentially capture light from multiple objects and might also suffer from pixel cross-talk, which leads to blurred or mixed depth measurements^39^. We also focus on challenging imaging scenarios in which the LiDAR sensor receives only a limited number of photons, due to long-range imaging or fast acquisition settings, and is affected by significant background noise, such as in bright or scattering environments. To enhance performance under these conditions, multiscale information has been widely utilized in SPL restoration algorithms. For a given pixel and scale, multiscale models aggregate photon detections from a predefined neighborhood structure (e.g., $3 \times 3 \times 3$ kernel), thereby increasing the photon count at the cost of reduced spatial resolution. This trade-off results in improved data quality in low-photon or high-background regimes. The construction of each scale corresponds to the convolution of the 3D density field with a 3D kernel considered Gaussian in what follows. We propose the following likelihood to jointly capture the effects of multiple sensors, multiple spatial scales (assuming *S* distinct scales), and inter-pixel cross-talk effects

2 $$\begin{aligned} {\boldsymbol{p}}_i^{s}(c_i^s) | {\boldsymbol{\Theta }}\sim \sum _{m=1}^{M} \alpha _m \mathscr {N}\left( {\boldsymbol{R}}_s^{T} {\boldsymbol{\mu }}_m, {\boldsymbol{R}}_s^{T} (\bar{{\boldsymbol{\Sigma }}}_m^{s} + {\boldsymbol{D}}){\boldsymbol{R}}_s\right) + \alpha _{M+1} \mathscr {B}^s({\boldsymbol{p}}_i^{s}) \text { subject to } \sum _{m=1}^{M+1} \alpha _m = 1, \end{aligned}$$

where the subscript *s* denotes the scale, the multiscale and convolution blur effects are absorbed by the covariance matrix thanks to the Gaussian properties, ${\boldsymbol{D}}$ is a diagonal matrix that accounts for the uncertainty in the 3D position resulting from the projection onto a pixel, and ${\boldsymbol{R}}_s$ is a binary diagonal matrix used to drop a dimension when handling 2D data. Assuming a high-resolution 2D image as the first modality (whose events are denoted ${\boldsymbol{p}}_i^{1}(c_i^1)$) and a LiDAR histogram cube observed at *S-1* different scales (treated as independent observations) denoted as ${\boldsymbol{p}}_i^{s}$ with $s\in \{2, \cdots , S\}$, the following observation model is considered:

3 $$\begin{aligned} {\boldsymbol{p}}_i^{1}(c_i^1) | {\boldsymbol{\Theta }}\sim & \sum _{m=1}^{M} \alpha _m \mathscr {N}({\boldsymbol{R}}_1^{T} {\boldsymbol{\mu }}_m, {\boldsymbol{R}}_1^{T} {\boldsymbol{\Sigma }}_m^{1} {\boldsymbol{R}}_1) + \alpha _{M+1} \mathscr {B}^1({\boldsymbol{p}}_i^{1}), \end{aligned}$$

4 $$\begin{aligned} {\boldsymbol{p}}_i^{s}(c_i^{s})| {\boldsymbol{\Theta }}\sim & \sum _{m=1}^{M} \alpha _m \mathscr {N}({\boldsymbol{\mu }}_m, {\boldsymbol{\Sigma }}_m^{s} ) + \alpha _{M+1} \mathscr {B}^2({\boldsymbol{p}}_i^{s}), \text { with } s \in \{2, \cdots , S\}, \end{aligned}$$

where we assume, without loss of generality, the histogram scales share the same background 3D density $\mathscr {B}^2$, and we simplify the covariance notation as follows ${\boldsymbol{\Sigma }}^s = \bar{{\boldsymbol{\Sigma }}}^s + {\boldsymbol{D}}, \forall s$. The multiscale covariance matrix is modeled as:

5 $$\begin{aligned} {\boldsymbol{\Sigma }}_m^s = {\boldsymbol{\Sigma }}_m^1 + \sigma _s^2 I, \forall s \end{aligned}$$

where the variables $\sigma _s^2, \forall s$ are the variances of the Gaussian multiscale kernels that are assumed known ($\sigma _s = k/2$ for a *k× k* kernel). This formulation offers robustness in the presence of noise and sparse photon regimes, owing to the multiscale processing strategy. It enables the fusion of 2D and 3D data within a unified framework and supports guided super-resolution at user-defined upsampling factors by learning a continuous representation of the scene. Moreover, it provides a compact data representation, as the scene at any resolution can be described using the parameters of the GMM.

**Fig. 9:**
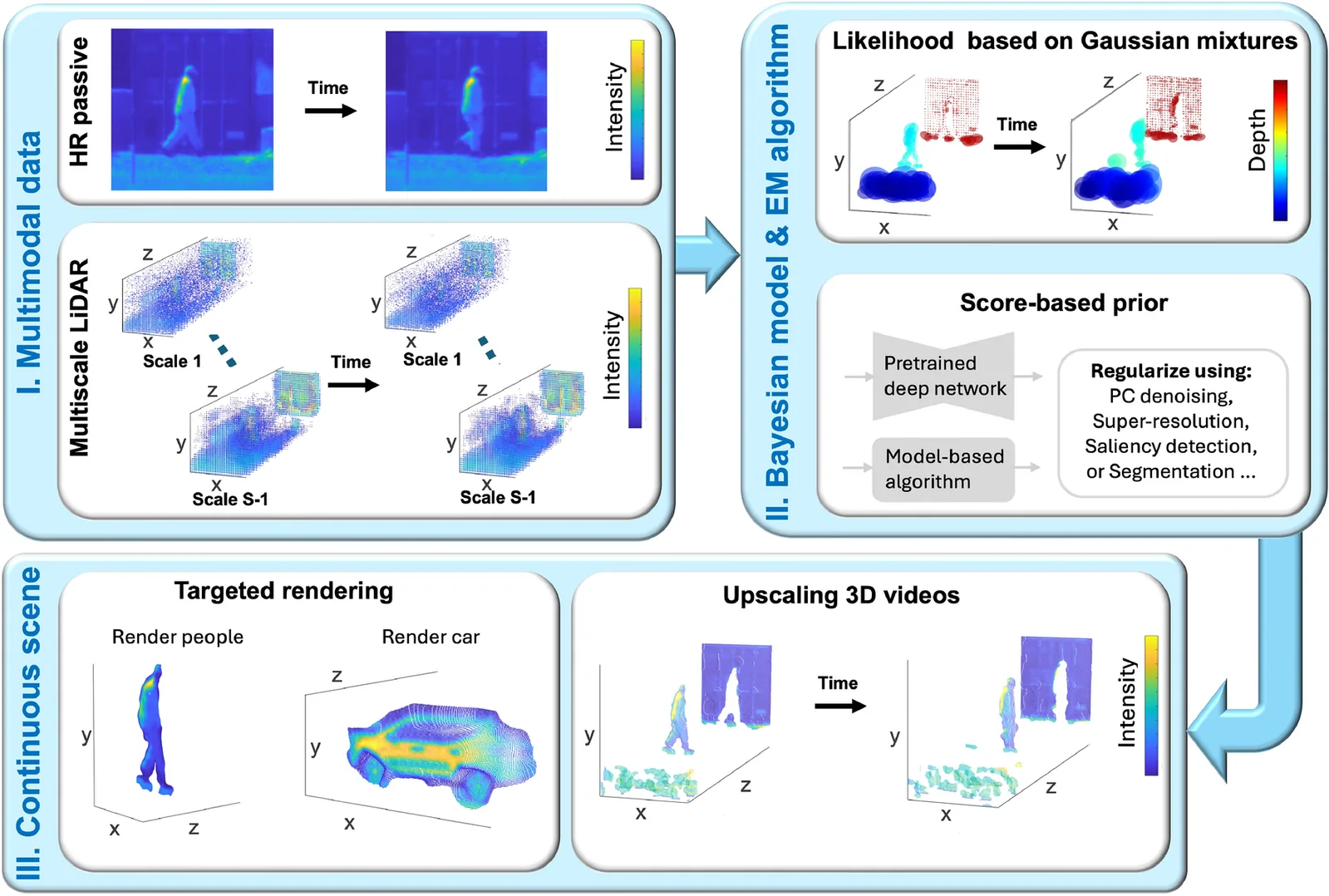
Block diagram illustrating the main steps of the continuous scene representation algorithm. The first block represents the input consisting of HR intensity frames and LiDAR data frames each evaluated at multiple scales (coarser scales are cleaner than the measured first scale). The second block illustrates the Bayesian inversion framework combining the GMM-based likelihood with score based priors, and solved using an expectation-maximization (EM) algorithm. The third block shows the resulting HR reconstructions of the full scene or regions of interest.

### Reconstruction algorithm

Our objective is to estimate the 3D density of the scene by recovering the parameters of the GMM. Estimating these parameters from the densities resulting from (3) and (4) captures all the information contained in the observations and corresponds to an MLE procedure. However, MLE is known to be sensitive to noise and limited data quality, which necessitates the use of additional regularization strategies. To address this, the reconstruction algorithm adopts a Bayesian framework that combines the data-driven likelihood $P({\boldsymbol{p}}_i^s \mid {\boldsymbol{\Theta }})$ with prior knowledge about the parameters of interest summarized in $P({\boldsymbol{\Theta }})$. This results in the following log-posterior distribution, which forms the basis for robust inference and reconstruction

6 $$\begin{aligned} \ell ({\boldsymbol{\Theta }}) = \sum _{s=1}^S \sum _{i=1}^{N_s} \log \sum _{z_i} P({\boldsymbol{p}}_i^s, z_i^s \mid {\boldsymbol{\Theta }}) + \log P({\boldsymbol{\Theta }}), \end{aligned}$$

where $z_i^s$ denotes a hidden label associating each detected photon to a term of the mixture, i.e. $z_i^s=m$ if the *i*th observation is associated with the *m*th Gaussian (as in^40^). The maximum a posterior parameter estimates can be obtained by maximizing (6). However, the presence of the summation inside the logarithm prevents a direct application to the distribution. A classical solution operates on the complete data log likelihood by considering the expectation-maximization (EM) algorithm that alternates between an expectation step to infer the hidden variables followed by a maximization step to estimate the mixture parameters ${\boldsymbol{\Theta }}^{(t)}$ at the *t*th iteration^40,41^, as follows:

7 $$\begin{aligned} & \text {E-step: } \,\, w_{im}^s \, = \, P(z_i^s=m | {\boldsymbol{p}}_i^s , {\boldsymbol{\Theta }}^{(t-1)} ) \end{aligned}$$

8 $$\begin{aligned} & \text {M-step: } {\boldsymbol{\Theta }}^{t} = \arg \max _{\boldsymbol{\Theta }}Q({\boldsymbol{\Theta }}, {\boldsymbol{\Theta }}^{(t-1)}) = \arg \max _{\boldsymbol{\Theta }}\left[ L({\boldsymbol{\Theta }}, {\boldsymbol{\Theta }}^{(t-1)}) + \log P({\boldsymbol{\Theta }}, {\boldsymbol{\Theta }}^{(t-1)}) \right] \nonumber \\ & \quad = \arg \min _{\boldsymbol{\Theta }}\sum _{s,i,m} \biggl \{ w_{im}^s c_i^s \log \alpha _m + w_{im}^s c_i^s \left[ ({\boldsymbol{p}}_i^s - {\boldsymbol{\mu }}_m)^T {\left( {\boldsymbol{R}}_s^{T} {\boldsymbol{\Sigma }}_m^{s} {\boldsymbol{R}}_s \right) }^{-1} ({\boldsymbol{p}}_i^s - {\boldsymbol{\mu }}_m) \right. + \log |{\boldsymbol{\Sigma }}_m^s | \Bigr ] \biggr \} - \log P\left( {\boldsymbol{\Theta }}, {\boldsymbol{\Theta }}^{(t-1)}\right) \end{aligned}$$

where $w_{im}^s$ is the responsibility of the cluster *m* to generate the data point *i* of scale *s*, *P*(.) denotes the prior distribution and $L (.) = \sum _{s=1}^S \sum _{i=1}^{N_s} \mathbb {E}_{z_i} \log P({\boldsymbol{p}}_i^s, z_i^s \mid {\boldsymbol{\Theta }})$ is the expected complete-data log-likelihood. The choice of the prior is crucial to regularize the inverse problem and enhance performance. Recent work has shown the benefit of using off-the-shelf denoisers as priors through plug-and-play schemes^21,42,43^. Taking the gradient of the terms in (8) leads to

9 $$\begin{aligned} \nabla _{{\boldsymbol{\Theta }}} L({\boldsymbol{\Theta }}, {\boldsymbol{\Theta }}^{(t-1)}) + \nabla _{{\boldsymbol{\Theta }}} \log P({\boldsymbol{\Theta }}, {\boldsymbol{\Theta }}^{(t-1)}) = 0, \end{aligned}$$

highlighting that the prior knowledge on the parameters ${\boldsymbol{\Theta }}$ of the GMM is incorporated through the “score” term, defined as $\nabla _{{\boldsymbol{\Theta }}} \log P({\boldsymbol{\Theta }}, {\boldsymbol{\Theta }}^{(t-1)})$. This gradient of the log-prior can be approximated using Tweedie’s formula as follows: $\nabla _{{\boldsymbol{\Theta }}} \log P({\boldsymbol{\Theta }}, {\boldsymbol{\Theta }}^{(t-1)}) \approx \frac{{\boldsymbol{\Theta }}- f({\boldsymbol{\Theta }}, {\boldsymbol{\Theta }}^{(t-1)})}{\sigma _f^2},$ where *f(· )* denotes the application of an off-the-shelf minimum mean square error (MMSE) estimator of ${\boldsymbol{\Theta }}$ and ${\sigma _f^2}$ its variance. This formulation allows the integration of existing model-based or data-driven algorithms as effective priors for the mixture parameters. In particular, it provides a practical and flexible mechanism for leveraging external estimators, whether data-driven or model-based, without requiring explicit prior distributions. This approximation is consistent with the approach in^42^, where the authors introduced a specific form of the prior distribution such that its gradient corresponds to the score function. The proposed inference strategy solves (9) for each mixture parameter using closed-form expressions derived within a fixed-point algorithm, detailed in the Supplementary Material. This versatile framework supports the incorporation of different priors tailored to the imaging scenario as illustrated in Fig. 9. In the experiments presented above, we demonstrate the use of two representative priors: a simple model-based point cloud denoiser for static scenes (involving outlier rejection followed by Gaussian smoothing), and a prior built using the deep-learning depth video super-resolution (DVSR) method^31^ for dynamic scenes. While the likelihood term models each frame independently, temporal consistency is implicitly enforced through the score-based prior, particularly when using pre-trained spatiotemporal priors such as DVSR. We also assess the algorithm’s performance across configurations, varying in particular the number of Gaussians used.

### Algorithms and evaluation metrics

We compare with multiple reconstruction algorithms that include the classical MLE for the Poisson observation model^16^, applied under the assumption of a single signal peak per pixel and a known background. In this approach, a matched filter based on the system’s impulse response is used to detect the time-of-flight peak position which is then refined using a center-of-mass approach^44^, and photon counts around the detected peak are integrated to estimate the intensity. Upsampled MLE results are obtained using the nearest-neighbor interpolation method on depth and intensity maps. The plug-and-play-based RT3D algorithm^21^ is also considered. This algorithm performs point-cloud reconstruction without relying on intensity guidance and can handle multiple peaks per pixel. However, it is not specifically designed for super-resolution tasks and is limited to an upsampling factor of *× 3*. To allow for a fair comparison, the histogram of counts is first upsampled using bilinear interpolation before being input to RT3D, thereby enabling reconstructions at the desired resolution. The third algorithm evaluated is the DVSR method^31^, which is an intensity-guided super-resolution approach. This method was pre-trained for an upsampling factor of *× 16*, and requires as input a high-resolution intensity image as guidance and an interpolated depth map that matches the size of the intensity input. To generalize DVSR to other upsampling factors, the input depth and intensity images are resized to the target resolution. While this may not be optimal for DVSR outside its original training configuration, it enables comparison with the other algorithms at the same target resolutions. The proposed algorithm can render a point cloud, or a depth map in presence of a single return peak per pixel. Therefore, we quantitatively evaluate its denoising and super-resolution performance using the depth absolute error DAE $= \frac{1}{N} \left| \left| {\boldsymbol{d}}^{\text {ref}}- {\boldsymbol{d}}^{\text {est}} \right| \right| _1$, where *N* is the number of pixels, ${\boldsymbol{d}}^{\text {ref}}$ and ${\boldsymbol{d}}^{\text {est}}$ are the reference and estimated depth maps, respectively.

**Human participants** Figs. 5 and 9 contain images of a human actor, who gave their full and informed consent for the data obtained to be used in reports and publications. All experimental protocols, trials plan, risk assessments and ethical considerations adhered to all national and international guidelines laid out by the UK Ministry of Defence, and were approved by Defence Science and Technology Laboratory (DSTL). All methods were carried out in accordance with relevant guidelines and regulations.

## Supplementary Information

Supplementary Information 1.

Supplementary Information 2.

## Data Availability

The datasets generated in the current study are available from the corresponding author upon reasonable request.
