# Supplementary material for: Continuous-surface 3D reconstruction from kilometer-range single-photon LiDAR using score-based priors
Source: Sci Rep. 2026 Jun 28;16:29663. doi: 10.1038/s41598-026-57483-5 (PMC13600943; doi:10.1038/s41598-026-57483-5)
Supplement: Supplementary file 1 [file 41598_2026_57483_MOESM1_ESM.pdf]

Supplementary Information

Continuous-Surface 3D Reconstruction from Kilometer-Range Single-Photon LiDAR using Score-Based Priors

A. Halimi, et al

Expectation-Maximization Estimation algorithm

The EM algorithm aims to optimize the equations (7)-(8) that are recalled here:

$$\text{E-step:}\;\; w^s_{im} = P(z^s_i = m | p^s_i, \Theta^{(t-1)})$$

$$\begin{aligned}\text{M-step: } \Theta^t &= \arg\max_{\Theta} L(\Theta, \Theta^{(t-1)}) + \log P(\Theta, \Theta^{(t-1)}) \\ &= \arg\min_{\Theta} \sum_{s,i,m} \left\{ w^s_{im} c^s_i \log \alpha_m + w^s_{im} c^s_i \left[(p^s_i - \mu_m)^T \left(R^T_s \Sigma^s_m R_s \right)^{-1} (p^s_i - \mu_m) + \log |\Sigma^s_m| \right] \right\} \\ &\qquad - \log P(\Theta, \Theta^{(t-1)}).\end{aligned}$$

- **E-step**

  The E-step evaluates w^s_{im} which represents the responsability of cluster m to generate the data point i of scale s:

$$\bar{w}^s_{im} = \frac{\alpha_m \mathcal{N}_{p^s_i}(\mu_m, R^T_s \Sigma^s_m R_s)}{\sum_{m'=1}^{M} \alpha_{m'} \mathcal{N}_{p^s_i}(\mu_{m'}, R^T_s \Sigma^s_{m'} R_s) + \alpha_{M+1} \mathscr{B}^2(p^s_i)} c^s_i = w^s_{im} c^s_i, \;\; \forall m \in \{1, \cdots, M\}$$

- **M-step**

  The M-step updates the GMM parameters μ_m, Σ_m, $\alpha_m, \forall m$ at fixed responsibilities, as detailed below.

  Updating $\mu_m, \forall m$

  The derivative of (1) with respect to μ_m in addition to the approximation of the score with the Tweedie formula leads to

$$\sum_{s,i} \left[2 w^s_{im} c^s_i (\Sigma^s_m)^{-1} (\mu_m - p^s_i) \right] + \sigma_\mu^{-2} [\mu_m - f_\mu(\mu_m)] = 0, \tag{1}$$

  and we propose to solve the fixed point equation. This leads to the analytical update

$$\sum_{s,i,m} \left\{ 2 w^s_{im} c^s_i (\Sigma^s_m)^{-1} (\mu^{\bar{t}}_m - p^s_i) \right\} + \sigma_\mu^{-2} (\mu^{\bar{t}}_m - f_\mu(\mu^{\bar{t}-1}_m)) = 0, \tag{2}$$

  where $\bar{t}$ denotes the iterations of the fixed point of (1), f_μ denotes the denoiser of μ and σ^2_μ its variance:

$$\mu^{\bar{t}}_m = \frac{R^T_1 \left(\Sigma^1_m\right)^{-1} R_1 \left[\sum_i \bar{w}^1_{im} p^1_i \right] + \sum_{i,s>1} (\Sigma^s_m)^{-1} \bar{w}^s_{im} p^s_i + \sigma_\mu^{-2} f_\mu(\mu^{\bar{t}-1}_m)}{R^T_1 \left(\Sigma^1_m\right)^{-1} R_1 \left[\sum_i \bar{w}^1_{im} \right] + \sum_{i,s>1} (\Sigma^s_m)^{-1} \bar{w}^s_{im} + \sigma_\mu^{-2}}. \tag{3}$$

  We fix $\bar{t} = 1$ in all experiments.

Updating $\alpha_m, \forall m$:
Enforcing the sum-to-one constraint and accounting for the prior $\log P(\alpha_m) = \bar{\alpha}_m \log \alpha_m$, where $\bar{\alpha}_m$ denotes the filtered version of this parameter, leads to: $\frac{\sum_{i,s} \bar{w}_{im}^s + \bar{\alpha}_m}{\alpha_m} - \lambda = 0$ where the sum-to-one constraint enforces $\lambda = \sum_m \left(\sum_{i,s} \bar{w}_{im}^s + \bar{\alpha}_m \right)$ leading to

$$\alpha_m = \frac{\sum_{i,s} \bar{w}_{im}^s + \bar{\alpha}_m}{\sum_m \left(\sum_{i,s} \bar{w}_{im}^s + \bar{\alpha}_m \right)}. \tag{4}$$

Updating $\Sigma_m^1, \forall m$:
The estimation of the covariances requires the minimization of the following expression:

$$\mathscr{L}(\Sigma_m^{-1}) = \sum_{i,s} \bar{w}_{im}^s \left((p_i^s - \mu_m)^T R_s^T \left(\Sigma_m^s\right)^{-1} R_s (p_i^s - \mu_m) \right) + \log |\Sigma_m^s| - \log P(\Sigma_m^{-1}).$$

Denoting $A_s = \bar{w}_{im}^s R_s (p_i^s - \mu_m)(p_i^s - \mu_m)^T R_s^T$ and considering the following properties

$$(\sigma_s^2 \left(\Sigma_m^1\right)^{-1} + I)^{-1} \approx I - \sigma_s^2 \left(\Sigma_m^1\right)^{-1}, \qquad \frac{\partial \mathrm{tr}(AXB)}{\partial X} = A^\top B^\top, \tag{5}$$

$$\left(\Sigma_m^s\right)^{-1} = (\Sigma_m^1 + \sigma_s^2 I)^{-1} = \left(\Sigma_m^1\right)^{-1} (\sigma_s^2 \left(\Sigma_m^1\right)^{-1} + I)^{-1}, \qquad \frac{\partial \log |X|}{\partial X} = X^{-T}, \tag{6}$$

to evaluate the derivative $\frac{\partial \mathscr{L}(\Sigma_m^1)}{\partial \Sigma_m^1} = 0$ leads to the following fixed point iterations:

$$\Sigma_{1,m} = \frac{\sum_s A_s - \sum_s \sigma_s^2 \left[\left(\Sigma_m^1\right)^{-1} A_s + A_s \left(\Sigma_m^1\right)^{-1} \right] + \sum_{i,s} \frac{\bar{w}_{i,m}^s \sigma_s^2}{2} \left(\Sigma_m^s\right)^{-1} \Sigma_m^1 + \left[\left(\Sigma_m^1\right)^{-1} - f_\Sigma \left(\left(\Sigma_m^1\right)^{-1} \right) \right] / \sigma_\Sigma^2}{\sum_{i,s} \frac{\bar{w}_{i,m}^s}{2}} \tag{7}$$

where we used $\nabla \log P(\Sigma_m^{-1}) = \left[\Sigma_{1,m}^{-1} - f_\Sigma(\Sigma_{1,m}^{-1}) \right] / \sigma_\Sigma^2$, with f_Σ the denoiser of Σ^{-1} and σ_Σ^2 its variance. We use 3 fixed point iterations in all experiments.

Computational cost

The computational time of the algorithm depends on several coupled factors, including the number of Gaussians M, the upsampling factor u_f, the number of pixels, the prior considered, and the noise/sparsity levels described by SBR and PPP. Since varying all these parameters jointly would lead to a prohibitively large study, we analyze representative cases using the same simulated data as in the main paper. To define the score prior, the proposed algorithm employs a simple model-based point cloud denoiser, applying outlier rejection (e.g., pcdenoise in MATLAB) and then Gaussian smoothing. Fig. S1(a) reports the computational time when varying M and u_f for reconstructing a 256×256 high-resolution scene from an input of size $256/u_f \times 256/u_f$. As expected, the time increases with M and is higher when processing larger input histograms, e.g., $u_f = 1$, compared with lower-resolution inputs, e.g., $u_f = 16$. Fig. S1(b) shows the effect of PPP and SBR using the normalized time ratio defined as

$$R_t = 1/5 \sum_{u_f \in \{1,2,4,8,16\}} \frac{\text{time}(\text{SBR}, \text{PPP}, u_f)}{\text{time}(\text{SBR} = 100, \text{PPP} = 1000, u_f)}.$$

The ratio is averaged over the upsampling factors, which showed similar trends. The results indicate that computation cost increases in photon-sparse or noisy regimes, reaching approximately twice the clean-case runtime in the most challenging settings.

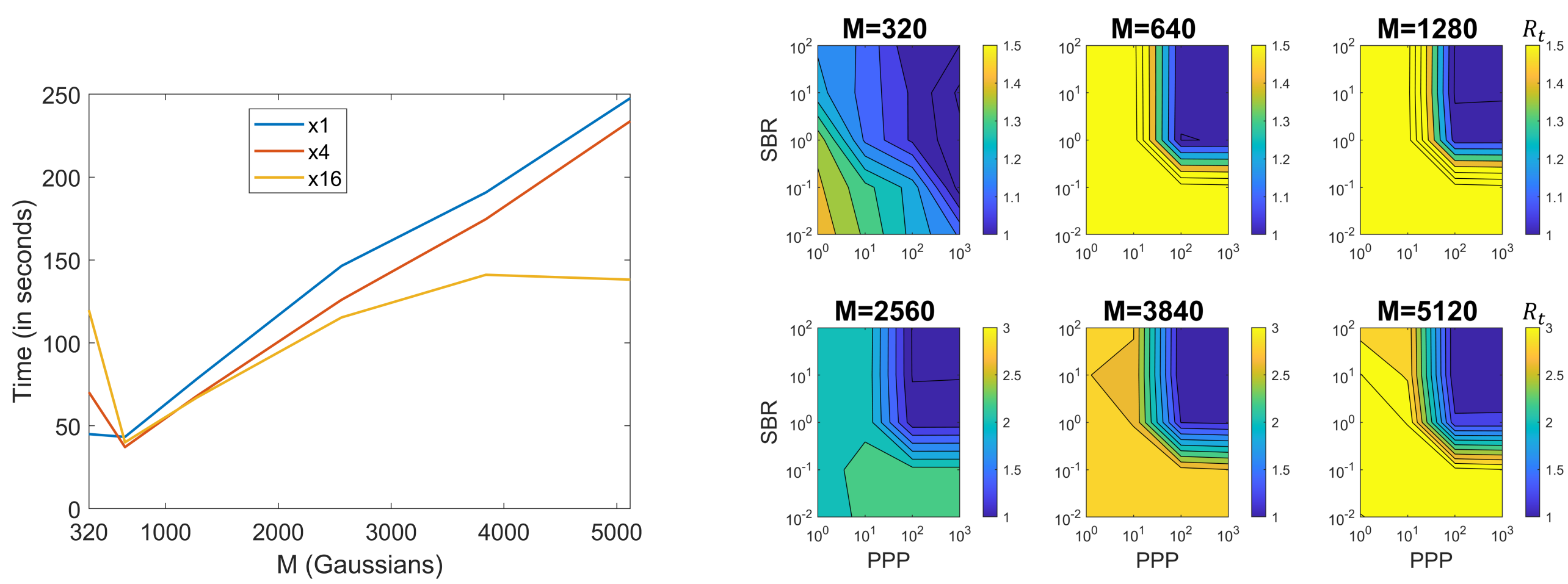

(a) **(b)**

Figure S1. Reconstruction time under varying resolution, number of Gaussians, sparsity, and noise conditions. (a) Reconstruction time (in seconds) of the proposed algorithm under clean conditions (PPP = 1000, SBR = 100), showing the effect of the upsampling factor u_f and the number of Gaussians M. **(b)** Normalized reconstruction time ratio R_t evaluated across different photon sparsity (PPP) and noise (SBR) levels. Results are shown for different values of M and averaged over u_f, since similar trends were observed across upsampling factors.

Background Estimation

The proposed approach assumes a known background distribution, denoted by $\mathscr{B}^2(p_i^{2:S})$. While the background is often modeled as uniform across the range dimension, it can exhibit non-uniform characteristics in scattering environments such as fog[1,2] or underwater scenes[3]. This section presents a method for estimating the background distribution by leveraging knowledge of the instrument response function (IRF) shape, as in[4], and assuming a spatially homogeneous 3D background, following the approach in[3]. Akin to[4], we begin by grouping the photon detection events into a histogram of counts for each pixel $y_n, \forall n$, and computing the Fourier transform along the range (temporal) dimension. Taking into account the instrument response function (IRF) spectrum, the resulting Fourier spectra are used to estimate the spectral densities of both the signal and background components. Fig. S2 presents a typical histogram of counts and its frequency spectrum for a photon-dense pixel. In such cases, the signal spectrum is confined within the spectral bandwidth of the IRF, while the background exhibits a smooth low-frequency component and a high-frequency component that reflects its variance. To estimate these contributions, the background variance is computed as the average spectral power over the high-frequency interval $[f_2, T/2]$. The signal spectrum is estimated from the frequency range $[f_1, f_2]$, after removing the background variance where f_1, f_2 are empirically selected based on the IRF spectral bandwidth (see Fig. S2 (right)). In what follows, we selected $f_1 = \frac{T}{2\pi\sigma}$, and $f_2 = 3.5f_1$, where σ is estimated from the full width at half maximum (FWHM) of the IRF, assuming a Gaussian shape. Finally, the remaining low-frequency component of the background is obtained by subtracting both the signal contribution and background variance from the total spectrum. These estimates are generally reliable in photon-rich conditions, as commonly observed in low-resolution histograms. In photon-sparse regimes, however, a low-pass spatial filter is applied to the histogram of counts to stabilize the spectral estimation. The estimated spectra are then used within

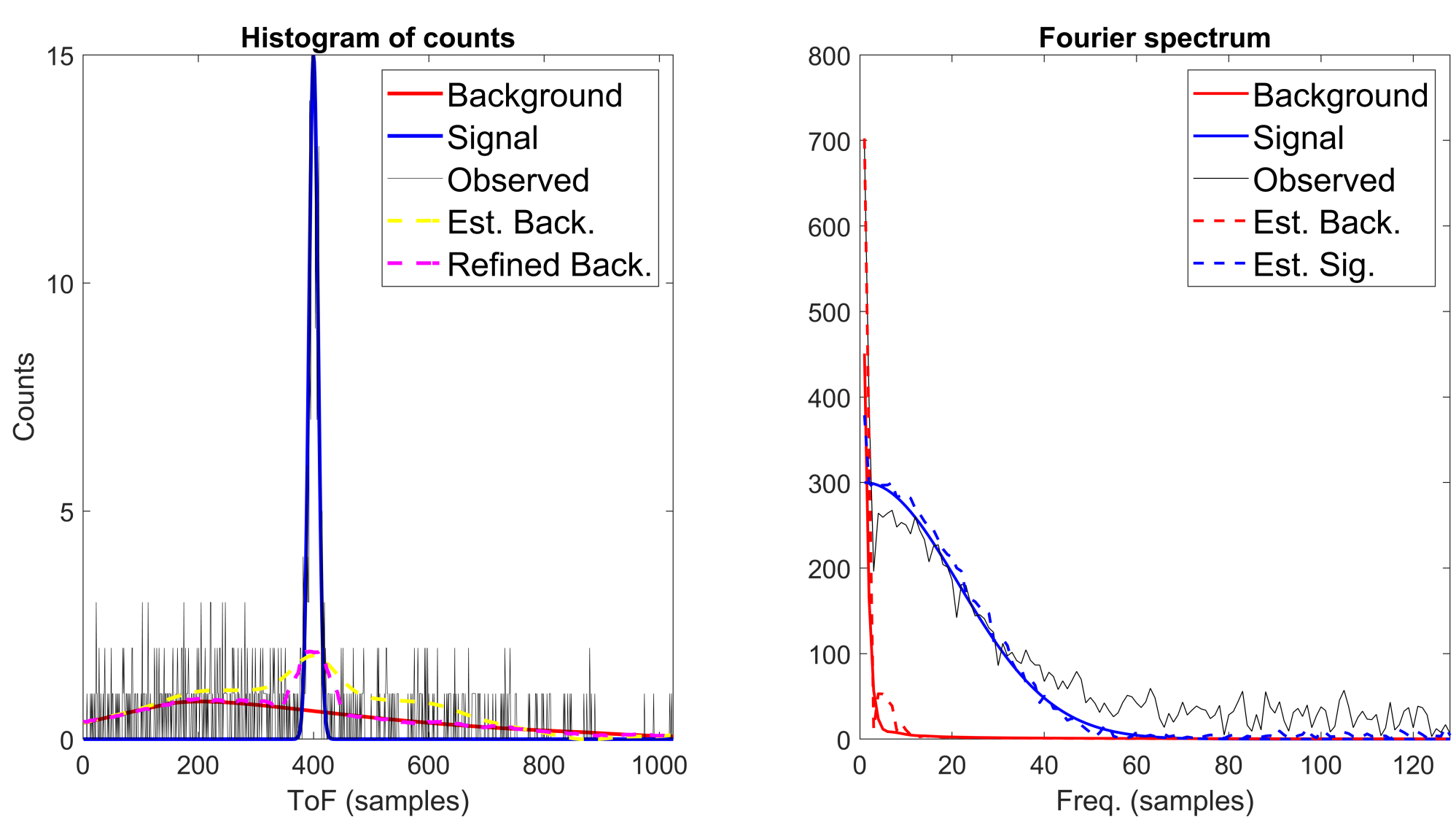

Figure S2. Example of a histogram of photon counts for a photon-dense pixel (PPP $= 620$, SBR $= 1$) and its corresponding frequency spectrum. **(Left)** Observed histogram of counts (black), with background (red) and signal (blue) components. The initial background estimate obtained via Wiener filtering is shown in yellow, and its refined version is shown in purple. **(Right)** Frequency spectra of the observed histogram (black), true background (solid red), and true signal (solid blue). The estimated background and signal spectra used in the Wiener filter are overlaid as dashed lines.

a Wiener filter to separate the signal s_n and background b_n components from the measured histogram

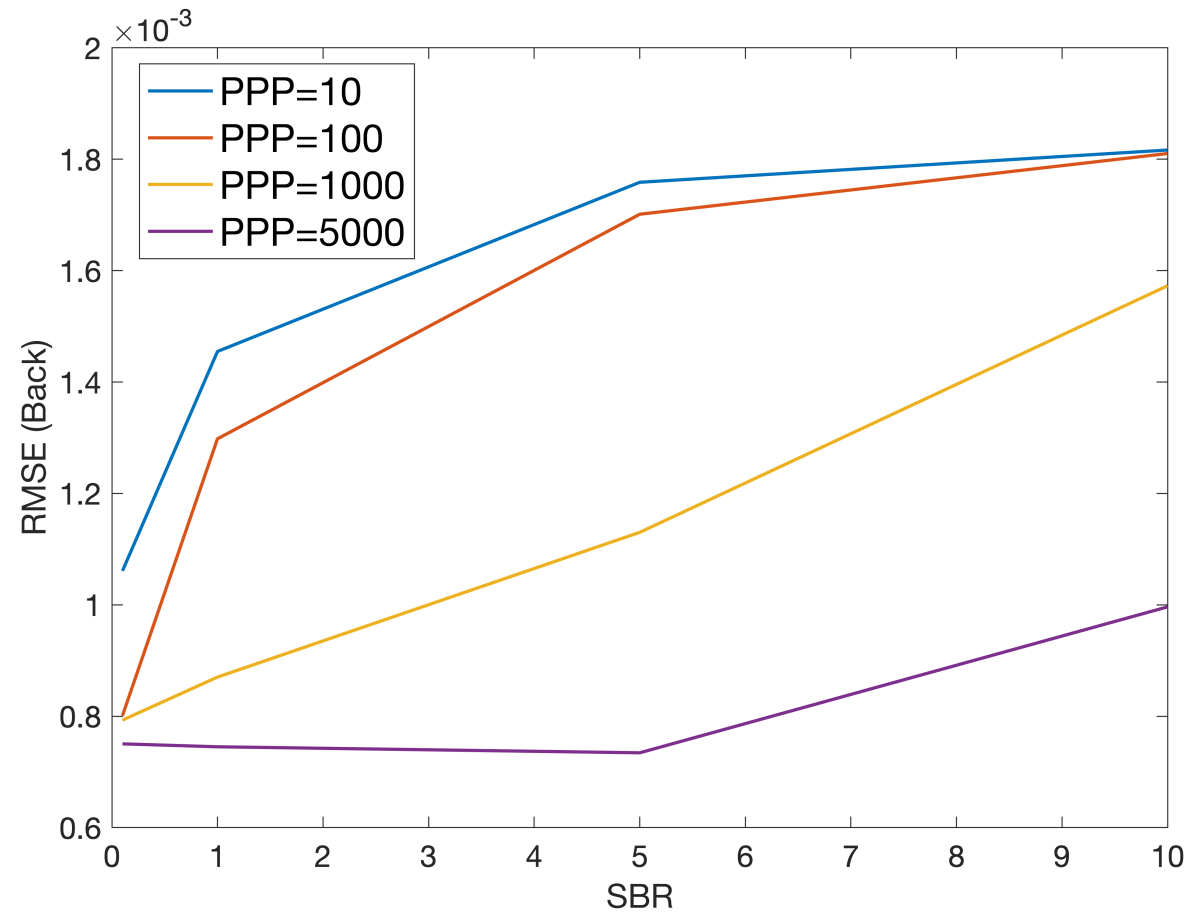

Figure S3. Performance of the proposed background estimation method for varying SBR and PPP values. The background has a gamma shaped distribution and the IRF is Gaussian with $\sigma = 8$.

y_n, providing an initial estimate of the background shape (see yellow curve in Fig. S2 (left)). This step leverages the IRF's spectral characteristics but does not exploit spatial correlations between neighboring pixels, which are important since background is often spatially smooth under homogeneity assumption. To refine this estimate, we perform a pixel-wise analysis on the histogram data. Signal counts are first detected by applying a thresholding rule: $(y_n - b_n) > 4\sqrt{b_n}$. The remaining counts are treated as background, and the resulting background histograms are then downsampled spatially to produce a smooth estimate of the full 3D background cube. This yields an estimate of the background distribution $\mathscr{B}^2(p_i^{2:S})$ (see purple curve in Fig. S2 (left)).

Figure S3 evaluates the performance of the proposed background estimation method across varying values of signal-to-background ratio (SBR), with SBR $\in [0.1, 10]$, and photons per pixel (PPP), with PPP $\in [10, 5000]$. We simulated histograms over a 32×32 pixel grid with $T = 512$ temporal bins, using a Gaussian IRF with standard deviation $\sigma = 8$, and per-pixel depth positions uniformly sampled from the interval $[50, 450]$. The background follows a gamma-shaped distribution, as illustrated in Fig. S2. Performance is quantified using the root mean square error (RMSE) between the estimated and true normalized background distributions. As expected, the results show improved accuracy in photon-rich regimes, with a decline in performance under sparse conditions. It is important to note that the input to this algorithm consists of photon-dense histograms. In practice, low-resolution histograms are typically photon-rich, and in sparse regimes, we apply a low-pass filter to increase the effective PPP and improve the background estimation.

Results at long range

The results of Fig. 6 of the main document are shown in Fig. S4 using depth-encoded colors to highlight differences among the four algorithms and to demonstrate the advantages of the proposed approach.

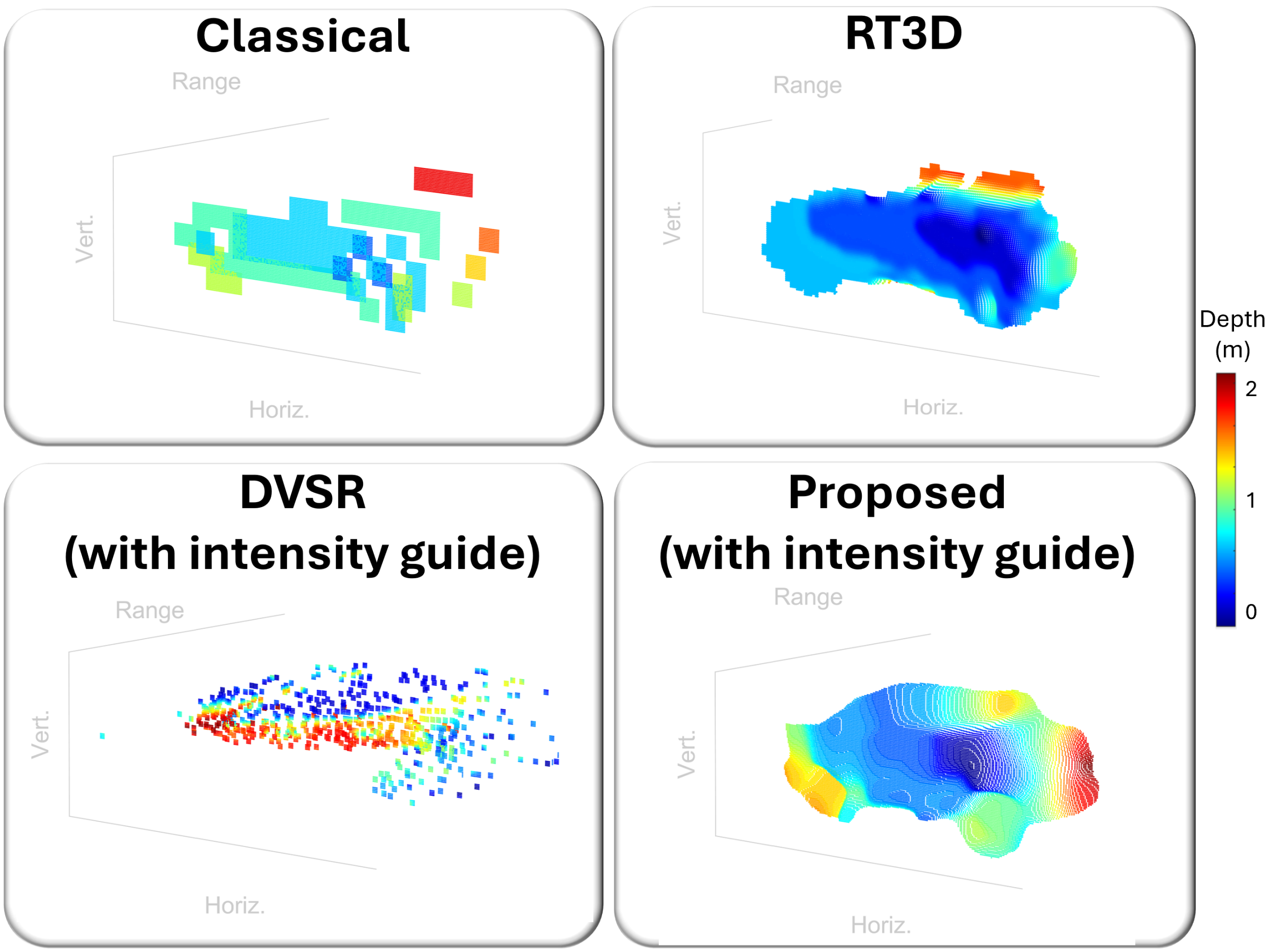

Figure S4. Upsampling results using classical nearest interpolation algorithm (top-left), RT3D algorithm (top-right), DVSR with intensity guidance (bottom-left) and proposed GMM algorithm with a DVSR-prior (bottom-right) for a car scene acquired at 1.4km stand-off distance from the LiDAR system. We show a ×16 upsampled. The colors encode the estimated depth values.

Algorithm details

Initialization: The EM algorithm requires a good initialization of the parameters Θ to accurately represent the 3D scene using the GMM. The multiscale approach[3] has proven effective in handling noisy or photon-sparse regimes. Our initialization procedure exploits it and consists of two main steps. First, using the estimated background shape and raw photon count data, we compute multiscale depth maps by applying a maximum likelihood estimator at each scale, for a defined number of peaks per pixel. We empirically set the number of peaks in proportion to the base-2 logarithm of the upscaling factor, reflecting the tendency of low-resolution histograms to exhibit more peaks due to spatial aggregation. These depth maps are then represented as a 3D point cloud, providing a reliable approximation of the signal counts. This point cloud is used to initialize Θ via a basic EM algorithm. If the resulting point cloud is significantly larger than the number of desired Gaussians M, an optional downsampling step is applied using K-means clustering to reduce it to approximately $10M$ points. In the presence of a 2D intensity guidance image, a second refinement step is performed, where the first two coordinates (x and y) are updated using an EM algorithm conditioned on the intensity map. In both steps, we ensure that the diagonal entries of the estimated covariance matrices are no smaller than the noise covariance D, maintaining numerical stability and modeling fidelity.

Number of Gaussians and scales: The number of Gaussians M is selected based on the desired compression ratio, as shown in Fig. 4 of the main paper. In practice, we found that $M = 1280$ offers a good trade-off for representing cluttered scenes with a single peak per pixel—such as the Middlebury Art scene—at a resolution of 256×256 pixels. The multiscale approach enhances robustness in noisy or photon-sparse regimes, where larger filtering kernels are beneficial. For scene denoising and reconstruction, we adopt default scales of 1×1, and 5×5, following the strategy in[3]. In the case of guided super-resolution, the low-resolution histogram of counts is typically photon-dense, so we use only the smaller scales of 1×1 and 2×2.

Implementation The EM algorithm was implemented in MATLAB R2024a and executed on a Windows 10 workstation equipped with an Intel(R) Xeon(R) Gold 6230 CPU @ 2.10 GHz and 192 GB RAM running on Windows 10. When deep priors were used, MATLAB called the corresponding Python implementation; in particular, DVSR was implemented in Python and executed on an NVIDIA GeForce RTX 3090 GPU with 24 GB memory.

Efficient implementation: During the expectation step, the contribution of all voxels is evaluated for each Gaussian in the mixture. Given the localized support of each Gaussian, an efficient implementation restricts computation to a 3D local neighborhood around each Gaussian center. Using smaller kernels improves computational efficiency but may degrade quality if the Gaussian contributions are not fully captured. This introduces a trade-off between the number of Gaussians M and the size of the local neighborhood: similar reconstruction quality can be achieved by increasing M while reducing the kernel size. In the paper's experiments, we adopt a default kernel size of $8 \times 8 \times 8sr$, where sr is the scale ratio modeling the size of an X-Y pixel relative to a temporal bin along the Z dimension.

Stopping criteria: The EM algorithm is iterative and requires appropriate stopping criteria. In this work, two criteria are used to terminate the iterations. The first is a predefined maximum number of iterations. The second monitors the convergence of the estimated parameters by computing the relative change between successive iterations; the algorithm stops when this change falls below a specified threshold.

Convergence: We note that the Tweedie-based score approximation does not provide a formal convergence guarantee for arbitrary external priors. To support this empirically, convergence curves in Fig. S5 show the normalized difference between successive reconstructed histogram cubes, z, obtained

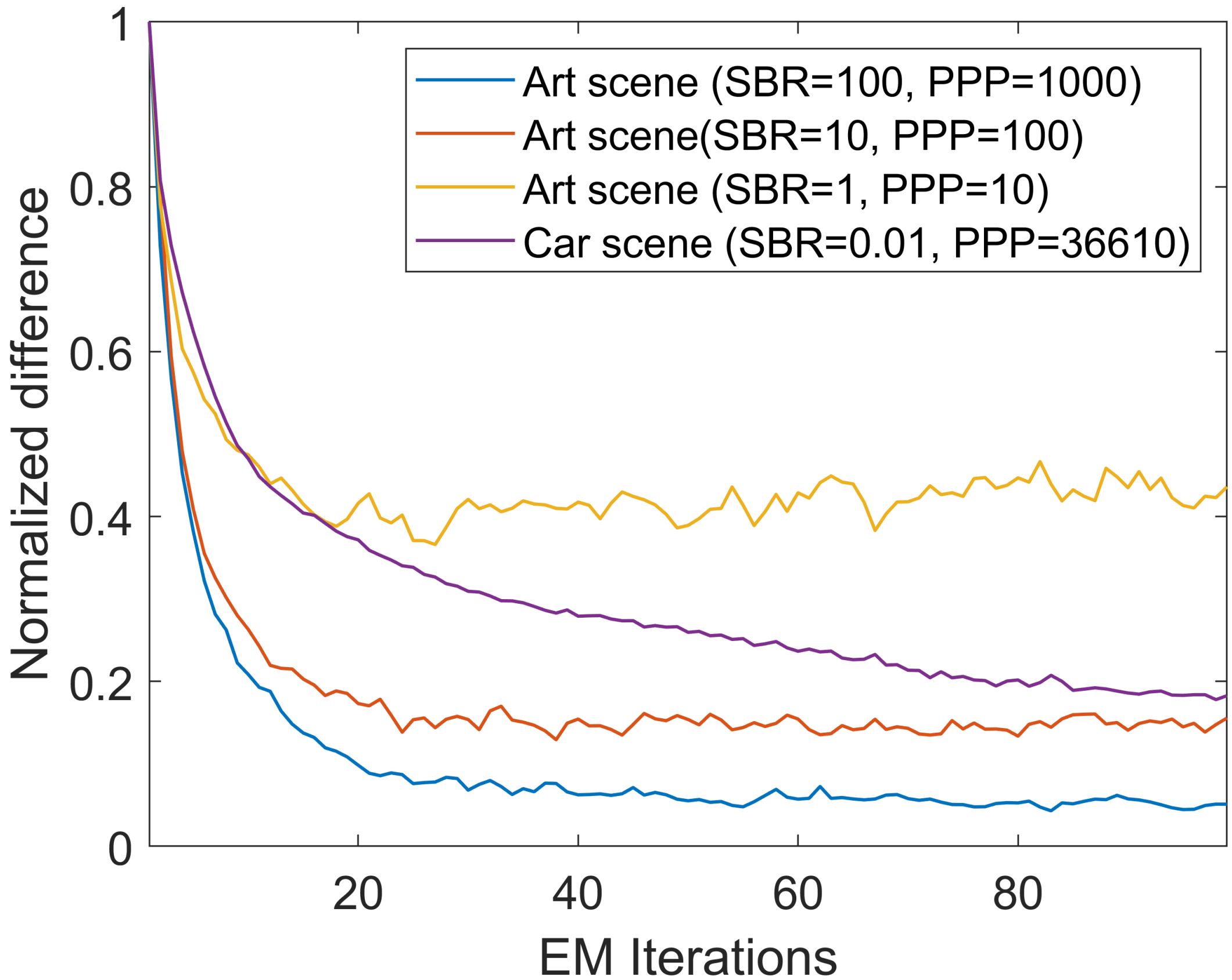

Figure S5. **Empirical convergence analysis of the proposed EM framework.** Evolution of the normalized difference between successive reconstructed histogram cubes, $\|z^{(t)} - z^{(t-1)}\| / \|z^{(2)} - z^{(1)}\|$, across EM iterations for representative simulated ($u_f = 1$) and real-data ($u_f = 4$) experiments. The curves indicate stable convergence behavior under different photon sparsity and noise conditions.

from the GMM parameters $\Theta = \{\mu_m, \bar{\Sigma}_m, \alpha_m\}_{m=1}^{M}$, i.e., $\frac{\|z^{(t)} - z^{(t-1)}\|}{\|z^{(2)} - z^{(1)}\|}$. The results show stable convergence for representative simulated and real-data examples.

References

1. Satat, G., Tancik, M. & Raskar, R. Towards photography through realistic fog. In *Computational Photography (ICCP), 2018 IEEE International Conference on*, 1–10 (IEEE, 2018).

2. Tobin, R., Halimi, A., McCarthy, A., Soan, P. J. & Buller, G. S. Robust real-time 3D imaging of moving scenes through atmospheric obscurant using single-photon lidar. *Sci. reports* **11**, 11236 (2021).

3. Halimi, A., Maccarone, A., Lamb, R., Buller, G. S. & McLaughlin, S. Robust and Guided Bayesian Reconstruction of Single-Photon 3D Lidar Data: Application to Multispectral and Underwater Imaging. *IEEE Trans. on Comput. Imaging* **7**, 961–974 (2021).

4. Lee, J., Ingle, A., Chacko, J. V., Eliceiri, K. W. & Gupta, M. Caspi: collaborative photon processing for active single-photon imaging. *Nat. Commun.* **14**, 3158 (2023).
